# User-centered design and related participatory approaches in digital health innovations for low-resource settings: A scoping review protocol

**DOI:** 10.1371/journal.pone.0337671

**Published:** 2026-09-08

**Authors:** Emmanuel Otukpa, Abdhalla Ziraba, Christopher Pell, Ben Ngoye

**Affiliations:** 1 Health Systems Strengthening Hub, Institute of Healthcare Management, Strathmore Business School, Strathmore University, Nairobi, Kenya; 2 Emerging and Re-emerging Infectious Disease Unit, Health and Wellbeing Theme, African Population and Health Research Center (APHRC), Nairobi, Kenya; 3 Department of Global Health, Amsterdam UMC, Location University of Amsterdam, Amsterdam, The Netherlands; 4 Amsterdam Public Health Research Institute, Global Health Program, Amsterdam, The Netherlands; 5 Amsterdam Institute for Global Health and Development (AIGHD), Amsterdam, The Netherlands; Pontificia Universidad Catolica de Chile, CHILE

## Abstract

**Objective:**

The primary objective of this scoping review is to identify and synthesize the existing evidence on the applications, reported outcomes, and key methodological considerations of user-centered design (UCD) and related participatory design methodologies including human centered design (HCD), co-design, participatory design and co-creation of digital health innovations within resource constrained settings.

**Introduction:**

A critical challenge in global health is the high failure rate of technologically sound innovations. This is due in part to insufficient consideration of the end-user’s environment, literacy levels, and socio-cultural beliefs. UCD is an iterative design process that grounds development in an understanding of the user’s needs, contexts, and feedback. It sits within a wider family of related approaches such as HCD, co-design, participatory designs and co-creation, while related to UCD, is distinguished by its emphasis on equitable partnership, where end-users are not merely informants but co-designers and co-developers of the innovation. These approaches have emerged as promising methodologies to develop more relevant, acceptable, and sustainable digital health solutions. In disease diagnostics, despite increasing interest, there is lack of a consolidated review mapping the full scope of how these methodologies are applied, across which types of digital health and diagnostic innovations, and with what outcomes.

**Inclusion criteria:**

Studies conducted in low-resource settings, published between 2015 and 2026 that apply UCD or a related participatory design methodology to the development, adaptation or evaluation of a digital health innovation and, where reported, outcomes such as usability, patient satisfaction, innovation success, adoption, scalability and sustainability. Studies that name such an approach without describing the activities undertaken are also eligible, and the completeness of reporting will be recorded and reported as a finding of this review.

**Methods:**

Guided by the JBI methodology for scoping reviews and reported in accordance with PRISMA-ScR, we will search MEDLINE, Embase, CINAHL, Scopus, Web of Science, IEEE Xplore, the ACM Digital Library, the WHO Global Index Medicus, and Google Scholar, together with named grey literature sources, for peer-reviewed articles, conference proceedings, and grey literature published in English and French from 2015 to 2026. Data extraction will include information on study characteristics, the design approaches and processes applied, the completeness with which they are reported as well as the outcomes.

## Introduction

A disproportionate number of preventable diseases, including both communicable and non-communicable diseases (NCDs), occur in low-resource environments worldwide [1,2]. Much of this burden is preventable or manageable with timely, accurate diagnosis and care, yet health-system constraints in infrastructure, financing, and workforce limit access to such care. These conditions range from communicable diseases, such as malaria, neglected tropical diseases, and zoonotic infections, to a rising burden of non-communicable diseases including cardiovascular disease, diabetes, and mental-health conditions [3].

These diseases are responsible for a substantial share of deaths worldwide, with the burden concentrated in low- and middle-income countries (LMICs) [3]. Part of this is a result of persistent health systems constraints in these settings, which is characterized by infrastructure, financial and human resource constraints that limit the adoption and impact of medical and diagnostic technologies analyses of health systems in sub-Saharan Africa document these constraints in detail [4].

In recent years, there has been a growing recognition of the potential of digital health technology to address healthcare concerns, in low-resource settings [5]. Telemedicine platforms enable remote consultations, allowing patients in underserved areas to receive medical advice without the need for long-distance travel [6]. Additionally, mobile health (mHealth) interventions leverage the widespread use of mobile phones to disseminate health information, send appointment reminders, and support medication adherence [7,8].

Digital health innovations span a wide range of applications relevant to low-resource settings. These include diagnostic tools such as smartphone-based microscopy and AI-assisted image analysis for malaria and schistosomiasis [9,10]; clinical decision support systems that assist providers in identifying antimicrobial-resistant organisms and selecting appropriate treatment regimens, with implications for antimicrobial stewardship [11]; digitally enabled point-of-care testing, which may help close diagnostic service gaps in underserved and remote areas [12–14]; and wearable sensors supporting continuous monitoring of chronic conditions, allowing a shift from episodic, clinic-based measurement towards remote monitoring and risk-based triage [15–19].

What these applications share is that their potential is contingent on use. Technical performance established under controlled conditions does not by itself determine whether a tool is adopted, used correctly, or sustained in the settings where it is deployed. It is this gap between demonstrated capability and realized benefit that motivates attention to how such technologies are designed.

The high failure rate of technologically sound innovations that are implemented without adequate attention to the end-user’s environment, literacy levels, and sociocultural attitudes is a key concern in global health. [9,20]. Health technologies, including diagnostic tools, developed for high-income settings often fail when transferred directly to low-resource contexts partly due to a mismatch with local needs, capacities, and cultural realities [20]. Non-adoption and abandonment are neither marginal nor random: they arise from the interaction between a technology and the organizational, infrastructural, and social conditions of its setting, and are therefore in principle, addressable at the design stage A paradigm shift is provided by User-Centered Design (UCD) and related participatory approaches, which move from a top-down technology transfer model to a collaborative process that actively incorporates end-users throughout the development lifecycle, including patients, community health workers, and local clinicians. [7,10].

UCD is an iterative design methodology that centers product development around users, ensuring that their requirements, preferences, and feedback influence each stage of the design cycle [11]. This process has been reported to enhance usability and to increases user satisfaction and engagement with health technologies [11,12]. Key principles of UCD include a deep understanding of user requirements, active user involvement in evaluations, and iterative testing and refinement of designs based on user input [13].

UCD belongs to a family of related methodologies that place users at the center of technology development: human-centered design (HCD), participatory design, co-design, co-creation, and design thinking. HCD is used largely synonymously with UCD, though often framed more expansively around the wider system of people, institutions, and social conditions surrounding a technology rather than the individual end-user alone [21]. Participatory design is grounded in a normative commitment to the right of those affected by a system to shape it, and co-design and co-creation extend this furthest, positioning users not as informants but as partners holding decision-making authority over the design of the innovation [12,14,22]. Working definitions and sources for each term are given in Table 1.

**Table 1:** Working definitions of user-involving design approaches used in this review.

Term	Working definition	Distinguishing emphasis	Key source
User-centred design (UCD)	Iterative design process in which users’ requirements, contexts, and feedback shape each stage of the design cycle.	Usability and fit to user needs; users as a source of requirements and evaluation.	Norman & Draper [23]; Gulliksen et al. [13]
Human-centred design (HCD)	Largely synonymous with UCD, but often extended to the wider system of people, institutions, and social conditions surrounding a technology.	Systemic framing beyond the individual end-user.	Göttgens & Oertelt-Prigione [21]
Participatory design	Design process grounded in the right of those affected by a system to influence its design.	Normative commitment to democratic participation.	Papoutsi et al. [24]
Co-design	Collaborative design in which users work alongside developers as active contributors to design decisions.	Shared authorship of design outputs.	Osterheider et al. [14]
Co-creation	Equitable partnership in which users hold decision-making authority as co-designers and co-developers.	Depth of partnership and shared control.	Hardyman et al. [22]
Design thinking	Structured problem-solving orientation, typically organised into discrete phases, within which the above methods are deployed.	Process architecture rather than a specific mode of user involvement.	Chen et al. [25]

What unites these approaches is iterative development grounded in users lived contexts; what distinguishes them is chiefly the depth of user involvement, which ranges along a continuum from consultation to equitable partnership. In practice the labels are applied inconsistently and often interchangeably [21,26]. It is for this reason that this review is framed around the family of approaches as a whole, rather than around any single named method, and that eligibility is determined by the user-involvement activities a study describes rather than by the term it uses. Throughout this protocol we use “UCD and related participatory design methodologies” to denote this family and reserve the specific terms for instances where a specific approach is meant. Working definitions are summarized in Table 1.

By emphasizing user experiences and actively involving patients or clients in the design process, these approaches may result in solutions that are not only practical but also accessible and egalitarian [14,24]. During the COVID-19 pandemic, healthcare organizations utilized human-centered design (HCD) approaches to rapidly develop and adapt digital tools aimed at improving access to care for underserved populations [7,27]. Such accounts illustrate how these approaches have been applied to support timely interventions and better resource allocation during health crises, although the evidence for their effect on outcomes remains dispersed and inconsistently reported.

### Rationale for the review

Implementing digital health innovations in LMICs is made more difficult by the absence of a thorough map of the models, processes, and reported results of UCD and related participatory design methodologies as they are applied in these contexts.

These settings present distinctive realities, including constrained infrastructure, heterogeneous digital and health literacy, intermittent connectivity, and diverse socio-cultural and linguistic contexts; that strongly shape whether digital health and diagnostic innovations are adopted and sustained. UCD and related participatory approaches are well suited to navigating precisely these realities, because they ground design in users’ lived contexts and constraints rather than in assumptions imported from high-resource settings. What remains unclear is therefore not whether these approaches can be applied in low-resource settings, but how they are applied: which methods, at which stages of development, and with which stakeholders and with what reported outcomes. This evidence remains scattered and has not been consolidated. Current literature indicates that while examples of participatory practice exists, such as participatory design initiatives in rural settings. A concrete and widely applicable framework for integrating these approaches in such contexts remains underdeveloped [23].

Without systematic documentation of how these approaches are applied and with what results, it is difficult to identify transferable practice, to replicate approaches that have worked elsewhere, or to judge which elements of a design process account for reported outcomes. Mapping this evidence is a precondition for any of these.

We include the full family of user-involving design approaches rather than a single named method because the terminology is used inconsistently across this literature: studies applying substantively similar methods describe them variously, and studies invoking the same label may describe very different depths of user involvement. Restricting the review to one or two labels would therefore fragment a body of evidence that is methodologically continuous and would systematically miss relevant work.

Within digital health, this review gives particular attention to diagnostic applications, reflecting both the persistent diagnostic gap in low-resource settings and the focus of the wider DI-DIDA project under which this review is conducted. Diagnostic applications are treated as one category of digital health innovation rather than as a separate scope.

### Objectives

The core objective of this review is to map the evidence on UCD and related participatory design methodologies in the development of digital health innovations, focusing on how these approaches are applied and what outcomes are reported in low-resource settings. A secondary objective is to characterize how completely these design processes are reported.

### Review questions

Through this review we will attempt to provide responses to the following questions:

1. What participatory and user-involving design approaches including UCD, HCD, co-design, participatory design, and co-creation and which named frameworks, processes, or models (e.g., the Double Diamond, IDEAS, the ISO 9241−210 cycle) are applied in the development of digital health innovations for low-resource settings, and how does their use vary across regions, healthcare contexts, and technology types?
2. How are participatory and user-involving design processes reported to influence the design and functionality of digital health innovations in low-resource settings at which stages of the development lifecycle, using which methods, with which stakeholder groups, and at what depth of user involvement?
3. What outcomes intended and unintended (e.g., usability, acceptability, adoption, clinical accuracy, scalability, sustainability) are reported in association with these approaches in digital health innovations in low-resource settings, and how are these outcomes measured and reported?
4. How completely are participatory design processes reported in this literature, and what proportion of studies invoke a named approach without describing the activities undertaken?

### Inclusion criteria

#### Population/setting.

For the purposes of this review, a low-resource setting is defined as one in which the design and deployment of digital health innovations is materially constrained by limitations in infrastructure, health workforce, financing, connectivity, or population-level digital and health literacy, or by structural barriers to healthcare access.

Studies are eligible if conducted in (i) a country classified by the World Bank as low-income, lower-middle-income, or upper-middle-income at the time the study was conducted; or (ii) a defined population or geographic area within a high-income country, where both of the following apply: (a) the study explicitly frames the innovation as responding to resource constraints or structural barriers to access affecting that population; and (b) the population corresponds to at least one externally defined designation of underservice or deprivation, as listed in S2 Table. Meeting criterion (b) alone is not sufficient: rurality or low income, without a stated resource-constraint rationale, does not render a study eligible. Studies of general populations in high-income countries are excluded. Where a study spans multiple settings, it is eligible if data relating to an eligible setting can be separately identified.

Eligible countries will be identified using the World Bank income classification for fiscal year 2020; the full country list is reproduced in Appendix I. Where a country’s classification changed during the study period, eligibility is determined by its classification at the time the study was conducted; where the year of conduct cannot be determined, the classification at the year of publication is applied and recorded during extraction.

Within these settings, the review focuses on studies that apply UCD or a related participatory design methodology to the development, adaptation, or evaluation of a digital health innovation, and that report, where available, outcomes such as usability, patient satisfaction, innovation success, adoption, scalability, and sustainability.

#### Context.

Digital health interventions, including but not limited to:

- Digitally enabled diagnostics and point-of-care testing (e.g., smartphone-based microscopy, AI-assisted image interpretation, connected or reader-based point-of-care tests, digital diagnostic decision support).
- eHealth (web-based applications).
- mHealth (mobile health applications).
- Telemedicine or telehealth platforms.
- Wearable health technologies
- Diagnostic tests without a digital component (e.g., conventional lateral-flow rapid diagnostic tests read by eye) are not eligible unless the innovation includes a digital element such as automated reading, image capture and analysis, connectivity, or data capture.

#### Types of studies.

This review aims to explore empirical studies (quantitative, qualitative, or mixed methods) and eligible grey literature, including conference proceedings, technical reports, and dissertations. Existing systematic reviews, scoping reviews, and meta-analyses will not be included as primary results; instead, their reference lists will be screened to identify relevant primary studies for inclusion, and they may be drawn on for background and contextual discussion.

To be eligible, a study must report the development, adaptation, or evaluation of a digital health innovation in a low-resource setting, and must meet at least one of the following: (a) it describes at least one concrete user-involvement activity (e.g., interviews, focus groups, contextual inquiry, participatory prototyping, co-design workshops, or usability testing) with end-users or other relevant stakeholders at one or more stages of development; or (b) it explicitly claims the use of a named user-involving design approach including UCD, HCD, co-design, co-creation, participatory design, or design thinking without describing the associated activities.

Studies meeting only criteria (b) are retained deliberately: the inconsistent and sometimes nominal use of this terminology is itself a recognized feature of the field, and quantifying its extent is an objective of this review. Each included study will be classified during extraction according to the completeness with which the design process is reported (fully described, partially described, or label-only), and this classification will be reported as a finding. Label-only studies will be analyzed as a distinct stratum and excluded from the synthesis of methods, stages, and depth of involvement, which they cannot inform.

Approaches applied to the design or development of a digital health innovation are eligible; participatory approaches applied solely to service delivery, health promotion, or research governance, with no associated technology, are not. Formal evaluation or outcome measurement is not required for inclusion, and the presence or absence of outcome reporting will be recorded during extraction.

#### Publication characteristics.

We will focus on English and/or French literature published in peer-reviewed journals or as grey literature from recognized organizations (e.g., WHO, government bodies). We will include studies published between 1 January 2015 and 31 December 2026; studies published outside this window will be excluded. This 11-year window is applied as a database-level limit at the search stage and is intended to capture the period of rapid scale-up of digital health and point-of-care diagnostic technologies, aligned with the era framed by the WHO Global Strategy on Digital Health (2020–2025), so that the evidence remains relevant to current technologies and design practice.

## Methods

This scoping review will be conducted in accordance with the JBI methodology for scoping reviews, with the overarching goal of mapping, clarifying, and identifying knowledge and methodological gaps, and will be reported in accordance with PRISMA-ScR and, for the search, PRISMA-S. The process involves a systematic approach to searching, screening, and reporting encompassing the following stages:: (1) identification of the research question (s); (2) identification of relevant databases and studies; (3) selection of studies; (4) data extraction; (5) interpretation, summarization and dissemination of the results. This approach is preferable as expected literature will report heterogenous methods and outcomes that may not be quantitatively pooled statistically into a single measure. The protocol has been registered on the Open Science Framework [25]

### Search strategy

Our search strategy aims to locate both published and unpublished studies. A three-step search strategy is being used. First, an initial limited search of MEDLINE (PubMed) and CINAHL (EBSCOhost) was conducted to identify articles on the topic. The text words contained in the titles and abstracts of relevant articles, and the index terms used to describe them, were used to develop the full search strategy, which is presented in Appendix I. Second, that strategy, including all identified keywords and index terms, has been adapted for each included database and information source. Third, the reference lists of all included sources of evidence will be screened for additional studies.

We will search for peer-reviewed and grey literature published in English and French between 1 January 2015 and 31 December 2025, without methodological restrictions. The databases and information sources to be searched are specified in Table 2, and the grey literature sources and the approach taken to each are specified in Table 3. The complete, untruncated search string for every source, together with the date on which it was run and the number of records retrieved, is reported in Appendix I in accordance with PRISMA-S. An overview of the search structure is presented in Table 4.

**Table 2:** Databases and information sources to be searched.

Database/ source	Platform or interface	Coverage searched	Rationale
MEDLINE	PubMed	2015-2026	Core biomedical literature
Embase	Elsevier	2015-2026	Broader European and pharmacological coverage
CINAHL	EBSCOhost	2015-2026	Nursing, allied health, and community health worker literature
Scopus	Elsevier	2015-2026	Multidisciplinary coverage, including conference output
Web of Science Core Collection	Clarivate	2015-2026	Multidisciplinary coverage and citation searching
IEEE Xplore	IEEE	2015-2026	Engineering and human-computer interaction literature
ACM Digital Library	ACM	2015-2026	CHI, DIS, COMPASS and ICTD proceedings, where much participatory design research with LMIC populations is published
WHO Global Index Medicus	WHO	2015-2026	Regional literature from LMICs (AIM, LILACS, IMEMR, IMSEAR, WPRIM) under-indexed in other databases
Google Scholar	Google platform	2015-2026	Supplementary searching; first 200 results screened

**Table 3:** Grey literature sources and search approach.

Source type	Specific sources	Approach
Preprint servers	medRxiv; arXiv (cs.HC)	Keyword search, 2015–2026
Theses and dissertations	ProQuest Dissertations & Theses Global; institutional repositories via BASE and CORE	Keyword search
Organisational repositories	WHO IRIS; World Bank Open Knowledge Repository; USAID Development Experience Clearinghouse; UNICEF	Repository search
Organisational websites	PATH; FIND; Digital Square; Global Digital Health Network	Structured browsing of publications sections
Conference proceedings	ACM CHI, DIS, COMPASS, ICTD; IEEE GHTC; AMIA	Covered via ACM Digital Library, IEEE Xplore, and Scopus
Targeted web searching	Google	First 100 results per search string, logged with date searched

**Table 4:** Search strategy: overview of facets (full strings for every database are given in S1 Table).

#1	“Community-based Participatory research”[MeSH] OR UCD OR “user-centered design”[tiab] OR “user centered design”[tiab] OR “human-centered design” OR “human centered design”[tw] OR “design thinking”[tw] ….
#2	“Digital Health”[Mesh] OR “Telemedicine”[Mesh] OR “Diagnostic Techniques and Procedures”[Mesh] OR “Point-of-Care Testing”[Mesh] OR “Medical Informatics”[Mesh] OR “Cell Phone”[tw] OR “Text Messaging”[tw] OR “digital health”[tw] OR “eHealth” [tw] OR “mHealth”[tw] …
#3	“Africa”[tw] OR “Asia”[tw] OR “South America”[tw] OR “Central America”[tw] OR “Afghanistan”[tw] OR “Bangladesh”[tw] OR “Benin”[tw] OR “Botswana”[tw] OR “Brazil”[tw] OR “Burkina Faso”[tw] OR “Burundi”[tw] OR “Cambodia”[tw] OR “Cameroon”[tw] OR “Chad”[tw] OR “China”[tw] OR “Colombia”[tw] OR “Congo”[tw] …
#4	Vulnerable Populations“[Mesh] OR “Medically Underserved Area”[Mesh] OR “Health Services Accessibility”[Mesh] OR “Poverty Areas”[Mesh] OR “Rural Population”[Mesh] OR “Transients and Migrants”[Mesh] OR “Refugees”[Mesh] OR “Indigenous Peoples”[Mesh] OR underserved[tiab] OR “under-served”[tiab] OR marginali*[tiab]…
#5	#3 OR #4
#6	#1 AND #2 AND #5

Grey literature searching follows the same date limits and eligibility criteria as the database searches, is conducted by a named reviewer, and is documented with the date searched and the number of records retrieved from each source. Grey literature will be reported as a distinct stream in the PRISMA-ScR flow diagram. Preprints are eligible where no peer-reviewed version exists; where both exist, the peer-reviewed version is retained and the preprint treated as a duplicate. Commercial product documentation and promotional material are not eligible. Records retrieved will be deduplicated in Zotero before screening.

### Study/Source of evidence selection

We will use Covidence to screen records, exporting included titles to Excel for analysis. Two researchers will independently assess articles for inclusion by screening titles, abstracts, and full texts. Where the two independent reviewers disagree on the eligibility of a paper, a third reviewer will adjudicate using the same inclusion criteria.

Before full screening, both reviewers will pilot the criteria on a calibration sample of records, with particular attention to the two criteria most open to interpretation: whether a setting meets the definition of a low-resource setting, and whether an innovation includes a digital component. Decision rules will be refined following the pilot, and inter-rater agreement for setting eligibility will be reported. Records excluded on language grounds will be counted and reported by language rather than discarded silently.

### Data extraction

We will use a standardized form (S3 Table) to extract relevant data from the studies included. The following will be extracted: administrative and bibliographic information, study characteristics & context, the nature and details of digital health innovation, the design approaches and processes applied, including the stated methodology, the stages of user involvement, the specific methods used, and the depth of user involvement; the completeness with which the design process is reported; and reported key findings.

### Data synthesis and presentation

We will analyze the data using descriptive statistics and thematic analysis, with results organized in tables and charts and presented into themes that reflect the review objectives. Our thematic analysis will identify patterns and trends in the application of UCD and related participatory design methodologies. Quantitative data, where applicable, will be summarized descriptively. We will also conduct a narrative synthesis of the barriers and facilitators to co-creation and UCD to integrate findings across studies, focusing on methods, outcomes, and identified gaps.

Reporting completeness will be described and cross-tabulated by publication year, region, publication type, and stated approach, and the author-applied label will be compared against the recorded depth of user involvement, so that terminological variation is reported as a finding rather than treated as a filter. Findings will be reported separately for studies conducted in low- and middle-income countries and for those conducted with underserved populations in high-income countries, and the principal descriptive results will be presented both including and excluding the latter.

Throughout, this review characterizes what studies report. Where relationships between design processes and outcomes are described, these are presented as reported by the original authors; the review does not assess the validity of these claims or establish causal relationships between design approach and outcome.

### Study status and timeline

At the time of this revised submission the review is in progress. This review will be revised and conducted in accordance with the methods described in this revised protocol. An initial search was executed on June 2026 across the databases outlined in this protocol except for the WHO Medicus, retrieving 1809 records.

Following peer review of this protocol, the search strategy and eligibility criteria were amended as recorded in Table 5. Because these amendments materially change what the search retrieves, a corrected search will be executed across all databases and information sources in September 2026, and all records will be screened against the revised eligibility criteria; no eligibility decision made under the superseded criteria will be carried forward without re-screening. Both the original and the corrected searches will be reported in the PRISMA-ScR flow diagram of the completed review.

**Table 5:** Amendments to the protocol following peer review.

Date	Section amended	Change	Rationale
09-08-2026	Title; Abstract; throughout	Scope reframed from “UCD and co-creation” to UCD and related participatory design methodologies; definitions added (Table 1)	Peer review: the framing excluded HCD, co-design, participatory design, and design thinking, which the search and eligibility criteria already admitted
09-08-2026	Title; Abstract; throughout	Paired phrase “digital health and diagnostics” removed; digital diagnostics treated as a sub-category of digital health, with a digital-component requirement	Peer review: diagnostics are a sub-area of digital health, and the pairing was applied inconsistently
09-08-2026	Review questions	All questions rewritten and aligned; causal phrasing replaced with “reported to influence”; reporting-completeness question added	Peer review: questions were mutually inconsistent and implied causal inference beyond the scope of a scoping review
09-08-2026	Inclusion criteria	Studies naming a design approach without describing the activities are now eligible; reporting completeness recorded and reported	Peer review: excluding such studies was inconsistent with the review’s own premise, and their prevalence is itself a finding
09-08-2026	Inclusion criteria	Operational definition of low-resource setting added, anchored to World Bank classification and to external designations of underservice for high-income settings	Peer review: rurality or underserved status does not by itself constitute a low-resource setting
09-08-2026	Search strategy	NOT block excluding high-income settings removed; underserved-population facet added; hand-built country list replaced with the Cochrane EPOC LMIC filter	Peer review: the exclusion would have removed eligible studies of underserved populations in high-income countries
09-08-2026	Methods	Definitive list of databases and named grey literature sources specified (Tables 2 and 3); PRISMA-S items reported	Peer review: three inconsistent database lists appeared in the manuscript and grey literature searching was not reproducible

We anticipate completing screening by December 2026, data extraction by January 2027, and reporting by April 2027. These projections allow for the larger volume of records expected under the broadened eligibility criteria and search strategy. Any deviation from this revised protocol occurring during the conduct of the review will be reported in the final publication, together with its rationale and an assessment of its likely effect on the findings.

### Strengths and limitations

Strengths. This review systematically bridges two distinct but highly relevant fields: participatory design methodologies and the implementation of digital health interventions in low-resource settings. By mapping this intersection, it will provide a foundational overview that is currently absent, identifying convergent principles and applications that can guide future research and funding. It will show where research is being conducted, with whom, and for which types of technology, and will identify gaps; under-represented regions, diseases, and stakeholder groups providing a strategic roadmap for future primary research. By recording the completeness with which design processes are reported, it will also generate the first systematic account of how far the terminology of participatory design in this literature corresponds to described practice.

Limitations. The core concepts of participatory design and of low-resource settings are defined and reported inconsistently across the literature. Because studies that invoke a named approach without describing it are eligible, the principal risk is now the converse: studies that use participatory methods without labelling them as such may be missed. We mitigate this through activity-level search terms and reference-list screening, but some omission is likely.

This review is restricted to literature published in English and French, reflecting the language capabilities of the review team. This restriction risks under-representing evidence from Spanish- and Portuguese-speaking Latin America, Lusophone Africa, and Arabic- and Chinese-language literature, and therefore introduces a potential geographic bias that is material given the review’s focus on low-resource settings. We partially mitigate this through the inclusion of the WHO Global Index Medicus, which indexes regional databases with English-language abstracts, and we will record and report the number of records excluded on language grounds, disaggregated by language.

We anticipate substantial heterogeneity across included studies in the technologies described, the design approaches applied and the depth of user involvement they entail, the outcomes reported and the instruments used to measure them, and the settings in which the work was conducted. This heterogeneity is itself an object of the review, but it limits the comparability of findings across studies and precludes any quantitative pooling or comparative statement about the relative effectiveness of different design approaches. The review’s contribution is accordingly descriptive: it maps the range and distribution of reported practice rather than establishing which approaches produce better outcomes.

Consistent with JBI guidance for scoping reviews, we do not undertake critical appraisal of included studies. The review therefore describes what is reported without assessing the methodological quality of the studies or the reliability of the outcomes they report; reported associations between design process and outcome are typically uncontrolled and frequently reported by the design teams themselves, which further constrains what can be inferred. Finally, the boundary between resource-constrained populations in high-income countries and the wider literature on underserved populations involves judgement; the criteria in Appendix I were specified in advance to constrain it, and findings will be presented both including and excluding these studies. Grey literature and Google Scholar searching are not fully reproducible, and results may vary by search date and location.

## Supporting information

S1 Table Search strategy. (DOCX)

S2 Table Externally defined designations accepted as evidence of a low-resource setting within a high-income country. (DOCX)

S3 Table Data collection instrument. (DOCX)

S4 Table PRISMA-P-checklist__protocol_review. (DOCX)
