# Supplementary material for: User-centered design and related participatory approaches in digital health innovations for low-resource settings: A scoping review protocol
Source: PLoS One. 2026 Sep 8;21(9):e0337671. doi: 10.1371/journal.pone.0337671 (PMC13552726; doi:10.1371/journal.pone.0337671)
Supplement: Supplementary file 1 [file pone.0337671.s001.docx]

*S1 Table Search strategy*

| #1 | "Community-based Participatory research"[MeSH] OR UCD OR "user-centered design"[tiab] OR "user centered design"[tiab] OR "human-centered design" OR "human centered design"[tw] OR "user-centred design"[tiab] OR "user centred design"[tiab] OR "human-centered design" OR "human centred design"[tw] OR "design thinking"[tw] OR co-design[tiab] OR "co design"[tw] OR co-creation[tiab] OR "co creation"[tiab] OR co-production[tw] OR "co production"[tw] OR "participatory design"[tw] OR "community engagement"[tw] OR "community involvement" OR "stakeholder engagement" OR "patient engagement" |
| --- | --- |
| #2 | "Digital Health"[Mesh] OR "Telemedicine"[Mesh] OR "Diagnostic Techniques and Procedures"[Mesh] OR "Point-of-Care Testing"[Mesh] OR "Medical Informatics"[Mesh] OR "Cell Phone"[tw] OR "Text Messaging"[tw] OR "digital health"[tw] OR "eHealth" [tw] OR "mHealth"[tw] OR "mobile health"[tw] OR "telehealth"[tw] OR "telemedicine"[tw] OR "health innovation*"[tw] OR "health technology"[tw] OR "diagnostic tool*"[tw] OR "point-of-care test*"[tw] OR "point of care test*" OR "medical app*"[tw] OR "mobile app*" OR "smartphone"[tw] OR "SMS"[tw] OR "text message"[tw] |
| #3 | "Africa"[tw] OR "Asia"[tw] OR "South America"[tw] OR "Central America"[tw] OR "Afghanistan"[tw] OR "Bangladesh"[tw] OR "Benin"[tw] OR "Botswana"[tw] OR "Brazil"[tw] OR "Burkina Faso"[tw] OR "Burundi"[tw] OR "Cambodia"[tw] OR "Cameroon"[tw] OR "Chad"[tw] OR "China"[tw] OR "Colombia"[tw] OR "Congo"[tw] OR "Costa Rica"[tw] OR "Côte d'Ivoire"[tw] OR "Djibouti"[tw] OR "Ecuador"[tw] OR "Egypt"[tw] OR "El Salvador"[tw] OR "Eritrea"[tw] OR "Eswatini"[tw] OR "Ethiopia"[tw] OR "Gabon"[tw] OR "Gambia"[tw] OR "Ghana"[tw] OR "Guatemala"[tw] OR "Guinea"[tw] OR "Haiti"[tw] OR "Honduras"[tw] OR "India"[tw] OR "Indonesia"[tw] OR "Iran"[tw] OR "Iraq"[tw] OR "Jamaica"[tw] OR "Jordan"[tw] OR "Kazakhstan"[tw] OR "Kenya"[tw] OR "Kuwait"[tw] OR "Kyrgyzstan"[tw] OR "Lao PDR"[tw] OR "Lebanon"[tw] OR "Lesotho"[tw] OR "Liberia"[tw] OR "Libya"[tw] OR "Madagascar"[tw] OR "Malawi"[tw] OR "Malaysia"[tw] OR "Mali"[tw] OR "Mauritania"[tw] OR "Mexico"[tw] OR "Mongolia"[tw] OR "Morocco"[tw] OR "Mozambique"[tw] OR "Myanmar"[tw] OR "Namibia"[tw] OR "Nepal"[tw] OR "Nicaragua"[tw] OR "Niger"[tw] OR "Nigeria"[tw] OR "Oman"[tw] OR "Pakistan"[tw] OR "Panama"[tw] OR "Papua New Guinea"[tw] OR "Paraguay"[tw] OR "Peru"[tw] OR "Philippines"[tw] OR "Rwanda"[tw] OR "Saudi Arabia"[tw] OR "Senegal"[tw] OR "Sierra Leone"[tw] OR "Somalia"[tw] OR "South Africa"[tw] OR "South Sudan"[tw] OR "Sri Lanka"[tw] OR "Sudan"[tw] OR "Syria"[tw] OR "Tajikistan"[tw] OR "Tanzania"[tw] OR "Thailand"[tw] OR "Timor-Leste"[tw] OR "Togo"[tw] OR "Tunisia"[tw] OR "Turkey"[tw] OR "Turkmenistan"[tw] OR "Uganda"[tw] OR "Ukraine"[tw] OR "Uzbekistan"[tw] OR "Vanuatu"[tw] OR "Vietnam"[tw] OR "West Bank"[tw] OR "Yemen"[tw] OR "Zambia"[tw] OR "Zimbabwe"[tw] OR "Developing Countries"[Mesh] OR "developing country"[tw] OR "developing countries"[tw] OR "developing nation"[tw] OR "developing world"[tw] OR "low resource setting"[tw] OR "low resource settings"[tw] OR "resource limited setting"[tw] OR "resource limited settings"[tw] OR "underdeveloped countries"[tw] OR "less developed countries"[tw] OR "low income country"[tw] OR "low income countries"[tw] OR "middle income country"[tw] OR "middle income countries"[tw] OR lmic[tw] OR lmics[tw] |
| #4 | "Vulnerable Populations"[Mesh] OR "Medically Underserved Area"[Mesh] OR "Health Services Accessibility"[Mesh] OR "Poverty Areas"[Mesh] OR "Rural Population"[Mesh] OR "Transients and Migrants"[Mesh] OR "Refugees"[Mesh] OR "Indigenous Peoples"[Mesh] OR underserved[tiab] OR "under-served"[tiab] OR marginali*[tiab] OR disadvantaged[tiab] OR deprived[tiab] OR "low-income"[tiab] OR "resource-limited"[tiab] OR "resource-constrained"[tiab] OR "resource-poor"[tiab] OR "low-resource"[tiab] OR rural[tiab] OR remote[tiab] OR refugee*[tiab] OR migrant*[tiab] OR indigenous[tiab] OR "informal settlement*"[tiab] OR slum*[tiab] |
| #5 | #3 OR #4 |
| #6 | #1 AND #2 AND #5 |

Eligible countries were identified using the World Bank income classification for fiscal year 2020 - 2021.
