# Supplementary material for: User-centered design and related participatory approaches in digital health innovations for low-resource settings: A scoping review protocol
Source: PLoS One. 2026 Sep 8;21(9):e0337671. doi: 10.1371/journal.pone.0337671 (PMC13552726; doi:10.1371/journal.pone.0337671)
Supplement: Supplementary file 2 [file pone.0337671.s002.docx]

*S2 Table Externally defined designations accepted as evidence of a low-resource setting within a high-income country*

| **Category** | **Accepted designations (examples)** | **Source to cite** |
| --- | --- | --- |
| Health workforce or service shortage | Medically Underserved Area / Population; Health Professional Shortage Area; equivalent national designations | HRSA (US) or the relevant national authority |
| Rural and remote classification | RUCA rural codes (US); Eurostat DEGURBA "thinly populated"; Modified Monash Model (Australia); national remote-area classifications | National statistical agency or Eurostat |
| Area deprivation | Most deprived quintile of a recognised national index (e.g. IMD, England; SIMD, Scotland; SEIFA, Australia; ADI, US) | Index publisher |
| Population group | Refugees; asylum seekers; undocumented migrants; Indigenous communities served by under-resourced health systems; people experiencing homelessness; incarcerated populations | UNHCR or the relevant national definition |
| Informal settlement | Areas meeting UN-Habitat informal settlement criteria | UN-Habitat |
