# Supplementary material for: User-centered design and related participatory approaches in digital health innovations for low-resource settings: A scoping review protocol
Source: PLoS One. 2026 Sep 8;21(9):e0337671. doi: 10.1371/journal.pone.0337671 (PMC13552726; doi:10.1371/journal.pone.0337671)
Supplement: Supplementary file 3 [file pone.0337671.s003.docx]

*S3 Table Data collection instrument*

| **Section A: Administrative and Bibliographic Information** | | | |
| --- | --- | --- | --- |
| # | Data Field | Description | Option |
| 1. | Research assistant | Name of the person performing the extraction | - Text input |
| 2. | Date of extraction | Date the extraction took place | - Dd/mm/yyyy |
| 3. | Publication Unique ID | A unique identifier for the study in the review (e.g., SR-001). | - Text input |
| 4. | Citation string | Full Citation in Vancouver | - Text input |
| 5. | Publication type | The type of literature | 1. Report (e.g. WHO, UN, World Bank) 2. Journal article, Conference 3. Proceeding 4. Thesis/dissertation 5. book chapter 6. Other (Specify) |
| 6. | Year | Year the literature was published | Integer input |
| 7. | Funding source | Source of funding that produced the literature | Text as reported by the publication |
| 8. | Conflict of interest | Reported conflict of interest | Text as reported by the authors -yes or no or not reported |
| **Section B: Study Characteristics & Context** | | | |
| 9. | Study objective | Description of the primary objective of the publication | Text input description of the primary object |
| 10. | Study design | Design of the literature | 1. Case study 2. Pilot study 3. Feasibility study 4. Qualitative study 5. Quantitative study 6. Mixed-methods 7. Protocol 8. Review 9. Others (specify) |
| 11. | Country/Countries | Country source of the literature | List of all countries |
| 12. | WHO region | Specific region where the country lies | - African Region - Region of the Americas - South-East Asia Region - Eastern Mediterranean Region - Western Pacific Region - European Region |
| 13. | World Bank Income level | Income level as determined by the World bank | - Low income - Lower middle income - Upper middle income - Not specified |
| 14. | World Bank classification year applied | Fiscal year of the classification used to determine eligibility | - Integer input |
| 15. | Setting eligibility route | Basis on which the setting met the inclusion criteria | LMIC (World Bank classification)  High-income country, underserved population  Unclear |
| 16. | Designation met (high-income settings only) | External designation satisfied, per Appendix I Table 6, and the authors' verbatim resource-constraint rationale | Text input |
| 17. | Study setting | Specific setting of the literature | - Urban - Rural - Peri-urban - Remote - Multiple - Not specified |
| 18. | Healthcare setting | Specific level where innovation is deployed in the study | - Primary care - Community level - Secondary care - Tertiary care - Public health campaign - Other |
| **Section C: Digital Health Innovation (details)** | | | |
| 19. | Name of innovation/tool | This is the name of the innovation provided by the authors | Free text (e.g., "mDiabetes App," "AI-powered TB diagnostic"). |
| 20. | Primary function | Details the main function of innovation | Free text (e.g., "Patient education," "Disease diagnosis," "Treatment adherence monitoring," "Data collection for health workers") |
| 21. | Technology type | This describes the type of technological development | - Mobile app - SMS/Text message service - Web tool - EHR/Dashboards - Wearable Sensor - POCT or POC device - Imaging device with digital component - AI machine learning - Other |
| 22. | Target disease/condition | Details the condition or disease the innovation is associated with | Free text (e.g., "HIV," "Maternal Health," "Diabetes," "Malaria," "General Primary Care"). |
| 23. | End users | Details of the primary target of the innovation | Community health workers  Patients/clients  Doctors (General Physicians)  Doctors (Specialists)  Nurses  Pharmacists  Lab Technologists  Multiple groups  Others |
| **Section D: Design Approach & Processes** | | | |
| 24. | Methodology | Stated Methodology/Framework | Coded list: UCD / HCD / co-design / co-creation / participatory design / design thinking / CBPR / other (specify) / none stated. A parallel free-text field records the authors' verbatim wording. |
| 25. | Rational for the process | Stated Rationale for using UCD/Co-Creation | Free text (e.g., "To improve usability," "To ensure cultural appropriateness," "To foster local ownership"). |
| 26. | Reporting completeness | Extent to which the design process is described | Fully described (methods, participants and stages all identifiable)  Partially described (one or two identifiable)  Label-only (approach named, no activities described) |
| 27. | Stage of involvement during development | Stage of involvement during development | Needs Assessment / Problem Identification  Requirements Gathering Ideation / Conceptualization Prototyping Usability Testing Implementation / Piloting Evaluation / Feedback Scaling / Sustainability Planning |
| 28. | Specific UCD/Co-Creation Methods Used | UCD/Co-Creation Methods Used | - Interviews - FGDs - Surveys/Questionnaires - Observations (contextual enquiries) - Persona development - Journey mapping - Co-design work - Participatory prototyping - Think out loud protocols - A/B testing - Other |
| 29. | User involvement | Details the cadre of individuals involved in the process | Free text list (e.g., "20 patients," "5 nurses," "15 community health workers," "3 local software developers"). |
| 30. | Level of involvement | Details the level of involvement from #5 | - Consultation – users give feedback - Direct involvement – users work directly with innovation teams - Partnership and co-creation – users are equal decision makers - User control – user led development |
| Section E: Reported Outcomes and Key Findings | | | |
| 31. | Innovation outcomes | Reported outcomes related to the innovation | - Improved Usability / User-Friendliness Increased Acceptability / Satisfaction Improved Feasibility Improved Clinical Efficacy/Accuracy (for diagnostics) Increased Adoption/Uptake Improved Sustainability Cost-effectiveness No significant outcome reported |
| 32. | Process outcomes | Reported Outcomes related to the PROCESS | Empowerment of Users/Community Increased Trust Development of Local Capacity Identification of Unanticipated Barriers/Needs Challenges reported (e.g., time-consuming, resource-intensive) |
| 33. | Facilitators | Key facilitators of UCD/Co-creation | Free-text summary of factors that enabled the process. |
| 34. | Barriers | Key Barriers to UCD/Co-Creation | Free-text summary of challenges faced. |
| 35. | Unintended outcomes | Key outcomes not predicted | Free-text summary of outcomes that were not planned for or foreseen |
| 36. | Author conclusion | The conclusions of the author’s observations | Free-text summary of the authors' main conclusions. |
| 37. | Reviewer notes | Reviewer's Notes / Key Quotes | Free-text field for notable quotes or reviewer observations not captured elsewhere |
