# Supplementary material for: User-centered design and related participatory approaches in digital health innovations for low-resource settings: A scoping review protocol
Source: PLoS One. 2026 Sep 8;21(9):e0337671. doi: 10.1371/journal.pone.0337671 (PMC13552726; doi:10.1371/journal.pone.0337671)
Supplement: Supplementary file 5 [file pone.0337671.s005.docx]

Response to reviewers

We thank the reviewer for the careful and constructive evaluation of our protocol. The comments have substantially improved the clarity, internal consistency, and methodological rigor of the manuscript. Below we respond to each comment on a point-by-point basis. The reviewer’s comments are shown in italics; our responses and the corresponding changes follow. All changes are visible as tracked changes in the revised manuscript, and line/section references point to the revised version. Where addressing a comment also resolved a latent inconsistency elsewhere in the protocol, we have noted this.

***** Summary of cross-cutting changes**

In addition to the point-by-point changes below, the revision resolved several internal inconsistencies that improve overall coherence: the eligibility window is now a single firm 2015–2025 range used everywhere; the framework attribution is corrected and PRISMA-ScR reporting is stated; the duplicated synthesis section was removed; the introduction’s disease framing and examples were broadened to match the disease-agnostic scope; the search terminology in the introduction, methods overview, and Appendix I is now mostly consistent; and the extraction instrument was corrected (income-level options) and extended (named design framework; basis for low-resource classification). We believe these revisions address the reviewer’s concerns in full and have strengthened the protocol considerably. We thank the reviewer again for their time and insight.

***Reviewer comment 1***

***The framing and motivation for the scoping review is not clear. In particular, in the introduction, it is not consistently clear that you are focusing on identifying papers that use UCD to design digital health interventions in low-resource settings. You mention digital health early on but then switch to talking about UCD and low-resource settings without digital health.***

**Response:** We agree that the introduction did not consistently hold the three core concepts; UCD/co-creation, digital health and diagnostics, and low-resource settings in view simultaneously, drifting into a generic account of UCD. We have rethreaded the introduction so the specific intersection that defines the review is foregrounded throughout, re-anchoring the UCD and co-creation paragraphs to digital-health/diagnostic examples in low-resource settings, and adding an explicit scope statement.

**Action taken:** Revised the Introduction and Rationale to re-anchor each design paragraph to digital health/diagnostics in low-resource settings and added an explicit closing scope sentence specifying that the review focuses on studies applying UCD/co-creation to the design of digital health and diagnostic innovations in low-resource settings. The objective and review questions were aligned to mirror this framing.

***Reviewer comment 2***

***The introduction/framing of the paper only mentions UCD and co-creation but there are many other related methodologies such as co-design, participatory design, human-centred design (HCD), etc. You use the term HCD in line 105 but did not introduce it. The paper would benefit from clearly defining the term(s) you plan to use, and explaining how they relate to other similar methodologies, and explaining in your search strategy how you will capture as many of these as possible.***

**Response:** We agree. We have added a short terminology paragraph defining the two organizing constructs we adopt (UCD and co-creation), naming the adjacent methodologies (HCD, design thinking, co-design, co-production, participatory design, community-based participatory research), and explaining how they relate as a partly overlapping continuum of user involvement. We introduce HCD explicitly at first use and clarify that our search deliberately captures the full terminological family.

**Action taken:** Added a terminology/conceptual-scope paragraph to the Introduction; introduced “human-centered design (HCD)” at first use (tracked change); and clarified in the Search strategy and Appendix I that Concept Block 1 is constructed to capture the full family of related terms regardless of the label authors use.

***Reviewer comment 3***

***Intro, line 115-119: You have not made a strong case for why UCD and co-creation methodologies would need to be applied differently in a low-resource setting. The factors you list here could impact the success of digital health interventions, but not UCD methods. In fact, I would argue that UCD methods help to overcome these types of challenges in digital health in low-resource settings.***

**Response:** We fully accept this point, which has strengthened our argument. We have rewritten the rationale so that contextual factors (infrastructure, literacy, connectivity, socio-cultural diversity) are no longer presented as obstacles to the methods, but as precisely the realities UCD and co-creation are suited to navigate. The genuine gap is relocated to the evidence base: the lack of a consolidated picture of how these methods are applied in low-resource settings and with what outcomes.

**Action taken:** Rewrote the Rationale passage (tracked change): the deleted clause framing contextual factors as impeding the methods is replaced with text positioning UCD/co-creation as well suited to these settings and locating the gap in the under-consolidated evidence on application and outcomes.

***Reviewer comment 4***

***Your third research question cannot be addressed with your planned methods. You would need to also identify studies that do not use UCD methods in order to compare them****.*

**Response:** The reviewer is correct. A comparison with non-co-creative approaches would require including comparator studies outside our inclusion criteria and beyond the descriptive intent of a scoping review. We have revised RQ3 to remove the explicit comparison and to focus on characterizing the outcomes reported within included UCD/co-creation studies, including how they are measured and which are sustained. Any within-study comparisons that appear will be summarized narratively.

**Action taken:** Revised RQ3 (tracked change) to: “What outcomes intended and unintended are reported in association with UCD and co-creation in digital health and diagnostic innovations in low-resource settings, how are these outcomes measured and reported?, and which are sustained over time?” The comparison clause was deleted.

***Reviewer comment 5***

***Methods: Your methods are not very clearly explained and are ambiguous in places. For example, the dates that will be included are not clear (some places say 10 year, some say 10-15). It would help to include an overview of the search strategy in the main paper, rather than the appendix (e.g., summarising the main search concepts and synonyms). It would also be helpful to have the databases that will be searched clearly listed (they are currently mentioned across different paragraphs, and a more detailed strategy is needed for Google Scholar which usually returns tens of thousands of results that cannot be systematically screened), as well as the inclusion/exclusion criteria. I would also suggest presenting your methods in the order they will be done. For example, you write that references of included articles will be screened before you have finished describing where the searches will take place. I see that you have checked these items off in the PRISMA checklist, but they did not feel very clear in the protocol.***

**Response:** We have revised the Methods comprehensively: standardized the eligibility window to 1 January 2015 – 31 December 2025; added a main-text overview of the three search concept blocks with representative synonyms (full strings in Appendix I); consolidated all databases and supplementary sources into a single list; specified a bounded, reproducible Google Scholar procedure; added explicit inclusion and exclusion criteria; and reordered the stages into execution sequence, moving reference-list/citation searching to after the database searches.

**Action taken:** Edited the abstract and Methods date language to a single firm window (tracked changes); corrected the framework attribution (see below); added the Types-of-studies/eligibility rewrite; and removed the duplicated synthesis section. Note: this comment also surfaced two latent inconsistencies we corrected the framework was mis-attributed (“JBI methodology... as updated by Levac et al.”; Levac et al. extends the Arksey & O’Malley framework, not JBI), now corrected to “JBI methodology for scoping reviews” with reporting per PRISMA-ScR; and the document contained two near-identical synthesis sections (“Data synthesis and presentation” and “Data analysis and presentation”), the duplicate of which has been deleted. Additionally we have included an overview of the search strategy within the text and specified the strategy for search for google scholar.

***Reviewer comment 6***

***Do they have to measure outcomes to be included? What if they only use co-design methods... but do not evaluate it?... What if they only mention the use of “UCD” or “co-design” but do not explicitly describe the methods used?***

**Response:** As a direct response, no, outcomes do not have to be measured. We have added explicit decision rules: (i) outcome measurement is not required for inclusion, since the review maps application; (ii) design-only studies are included, with the development stage recorded at extraction; (iii) the listed outcomes are illustrative of what we extract where present, not an inclusion threshold; and (iv) studies that merely name a label without describing any user-involvement activity are excluded, per a minimum-activity operational definition.

**Action taken:** Added the eligibility rules to the Types-of-studies/eligibility text (tracked change), including the minimum user-involvement-activity threshold and the explicit statement that formal evaluation is not required. The abstract inclusion wording was softened from “report on outcomes” to “where reported, outcomes” to remove the contradiction.

***Reviewer comment 7***

***Intro: in some areas, this protocol focuses on infectious disease specifically, though this does not seem to be part of the scoping review topic or strategy. I would suggest instead starting the introduction by talking about the burden of preventable diseases in low-resource settings, rather than listing rare infectious diseases of poverty.***

**Response:** We agree. We have rewritten the opening to motivate the review through the broader burden of preventable disease (communicable and non-communicable) and the systemic diagnostic/access gaps that digital health can help address. Specific infectious diseases are retained only as brief illustrations of digital diagnostic application, not as a statement of scope.

**Action taken:** Rewrote the opening paragraph (tracked change) to lead with the burden of preventable disease across communicable and non-communicable conditions and corrected the definitional error “non-infectious diseases (NCDs)” to “non-communicable diseases (NCDs).”

***Reviewer comment 8***

***Intro: Similarly, many of the digital health examples provided are very infectious-disease specific. If you plan to review studies that include all types of digital health tools, I think these examples should be expanded to include those that address health behaviours, chronic diseases, etc. as these are a huge area as well.***

**Response:** We agree and have broadened the illustrative examples to span non-communicable and chronic-disease management, health-behavior change and prevention, maternal and reproductive health, and mental health, alongside the existing infectious-disease diagnostic examples, presented explicitly as illustrations of the breadth the review will map.

**Action taken:** Expanded the digital-health example paragraphs (reflected in the broadened Introduction framing). We also corrected the word-order error “innovations digital health” to “digital health innovations” (tracked change), and flag for the editor that reference [6] supporting a point-of-care accuracy claim is a non-peer-reviewed source we are replacing.

***Reviewer comment 9***

***Please clarify how you will you define “low-resource” or “underserved” in high-income countries as part of your inclusion/exclusion criteria.***

**Response:** We have added an operational definition with two pathways: (i) LMICs classified using the World Bank income classification for the study year; and (ii) resource-constrained or underserved populations/sub-national areas within high-income countries, evidenced by indicators such as rural/remote populations, designated medically underserved areas, displaced or migrant populations, indigenous communities, or author-defined underserved groups, with a materiality requirement and reviewer adjudication for ambiguous cases.

**Action taken:** Added the operational definition to the eligibility criteria; corrected the extraction instrument (Appendix II, Section B) income-level options, where “Lower middle income” was duplicated and “Low income” omitted the first instance is changed to “Low income” (tracked change) and added a field recording the basis for the low-resource/underserved classification.

***Reviewer comment 10***

***...it is not typical to include systematic/scoping reviews in your results, but instead to check their references for relevant studies.***

**Response:** We agree. Existing reviews are no longer included as primary results; their reference lists will be screened to identify relevant primary studies, and they may inform background/contextual discussion. This avoids double-counting underlying primary studies.

**Action taken:** Revised the Types-of-studies criterion (tracked change) to exclude reviews as records while retaining them for reference-list screening and background; added a citation-screening step after the database searches; and removed “Review” from the extractable study-design options in Appendix II.

***Reviewer comment 11***

***You mentioned a restriction of 10 years in your abstract but state... that you will just “prioritize” publications in 10-15 years but not restrict them... Please also choose between 10 or 15 years. If you decide to restrict the strategy, please provide a justification.***

**Response:** We agree on both points. We have removed all “prioritization” language and adopted a single firm window of 1 January 2015 – 31 December 2025 (a 10-year period), applied as a hard database-level limit and enforced consistently throughout, with a justification added.

**Action taken:** Replaced the “no strict restriction... prioritizing... last 10–15 years” sentence with a firm 2015–2025 inclusion rule and justification (tracked change), and aligned the abstract date language (“within the last 15 years” → “between 2015 and 2025”). All other date references were reconciled.

***Reviewer comment 12***

***Search strategy: The search strategy needs refinement. You are missing the other spelling of “human-centered design” and many digital health-relevant terms like electronic medical records, electronic health records, chatbot, etc.***

**Response:** We have systematically revised Appendix I to include British/American and hyphenation variants for every design term (including both spellings of “human-centered/centered design”) and substantially expanded the digital-health block with the missing concepts electronic health/medical records (EHR/EMR), clinical decision support, chatbot/conversational agent, artificial intelligence/machine learning, remote/digital monitoring, wearables/sensors, and digital/computational diagnostics, among others. We have added a statement that the strategy will be peer-reviewed using the PRESS checklist before execution.

**Action taken:** Revised Appendix I (Concept Blocks 1 and 2) to add spelling/hyphenation variants and the additional digital-health terms; corrected typographical errors in Block 1 (“percon-centered”, unmatched bracket) and inserted explicit Boolean operators between synonym groups; and added a PRESS peer-review statement to the Methods. The main overview now reflects the expanded terms.

***Reviewer comment 13***

***It would also be helpful to extract data on any specific framework that was used by the researchers (e.g., the Double Diamond, IDEAS framework, etc.). This also seems closely related to your first review question.***

**Response:** We agree and have added a dedicated extraction field for the specific named framework/model applied (distinct from the broad methodology), seeded with common options (Double Diamond, Design Thinking/d.school, ISO 9241-210, IDEAS, Stanford Biodesign, EBCD, CeHRes Roadmap, etc.) plus “Other (specify)” and “None named.” We have noted in the synthesis that the distribution of these frameworks will be analyzed to answer RQ1.

**Action taken:** Added the “Specific design framework/model used” field to Appendix II, Section D; sharpened RQ1 to reference frameworks/models with examples (tracked change); and linked the new field to RQ1 in the Data synthesis section.

***Reviewer comment 14***

***Ethics: scoping reviews do not require ethics approval.***

**Response:** We agree that a literature-based scoping review does not require research-ethics approval. We have clarified this in the Ethics and dissemination section and reframed the institutional and national approvals as program-level (DI-DIDA) context reported for completeness, while retaining the dissemination and protocol-amendment statements.

**Action taken:** Added a leading sentence to the Ethics section (tracked change) stating that the review does not in itself require ethics approval, with the SU-ISERC/NACOSTI approvals reframed as broader-project context.

***Reviewer comment 15***

***I think this question needs to include “in low-resource settings.” At Line 146***

**Response:** We agree and have added the scope clause. We also confirmed that all three review questions are now consistently bounded to low-resource settings and that the phrasing “low-resource settings” is used consistently (hyphenated) throughout.

**Action taken:** Added “in low-resource settings” to the review question at the indicated location (tracked change), hyphenated “low-resource” in RQ1, and confirmed RQ3 retained the setting clause.
