# Supplementary material for: User-centered design and related participatory approaches in digital health innovations for low-resource settings: A scoping review protocol
Source: PLoS One. 2026 Sep 8;21(9):e0337671. doi: 10.1371/journal.pone.0337671 (PMC13552726; doi:10.1371/journal.pone.0337671)
Supplement: Supplementary file 6 [file pone.0337671.s006.docx]

We thank the Editor and the three Reviewers for the time and care taken with this protocol. The comments were detailed and constructive and addressing them has led to substantive improvements in the coherence and reproducibility of the review in particular to the framing of the design methodologies under study, the internal consistency of the scope statement, the eligibility criteria, the search strategy, and the accuracy with which we report the status of the work.

Below we respond to each of the comments in turn. Reviewers' comments are reproduced verbatim; each response is followed in blue by a description of the corresponding changes to the manuscript. Revised text is quoted where it aids review. In the manuscript itself, all changes are marked using tracked changes. Where two or more Reviewers raised related points and in one instance where their recommendations pulled in opposite directions we have addressed them together and flagged the connection, so that the coherence of our revisions can be assessed across the reviews as a whole.

Reviewer #1: The authors have responded to many of my earlier comments and the manuscript is improved. However, there are still some remaining issues that should be addressed for this protocol to be clearly reported, rigorous, and replicable. Please see below for my additional feedback:

1. Line 59: Given the global focus of the review, the frequent mentions of SSA in the Intro don’t make sense. I would suggest moving away from SSA and describing challenges across LMICs more broadly.

Thank you for this keen observation. On a re-examination, we do fine the framing of the Introduction inconsistent with the scope of the review, which is defined at the level of low-resource settings rather than any single region. The repeated references to sub-Saharan Africa were a legacy of the regional focus of the parent DI-DIDA project and were not intended to narrow the review.

We have revised the Introduction to describe the disease burden, health-system constraints, and diagnostic gaps across low- and middle-income countries and other low-resource settings more broadly. Sub-Saharan Africa is retained only where a cited source reports region-specific data, and in those instances, it is presented as an illustrative example rather than as the framing of the review. We have also checked the Rationale and Inclusion criteria to confirm that the population and setting framing is now consistent throughout. Specifically;

- Introduction, paragraph 2: burden and health-system text re-cast at LMIC level. Revised text reads:

“These conditions account for a substantial share of deaths worldwide, with the burden concentrated in low- and middle-income countries (LMICs) [3]. This is partly a consequence of persistent health-system constraints in these settings — including infrastructure, financing, and human-resource limitations — that restrict the adoption and impact of medical and diagnostic technologies [4].”

- Point-of-care testing paragraph: both references to sub-Saharan Africa replaced with “low-resource settings” and “these settings”; the sentence fragment beginning “Ensuring the relatively accurate testing…” merged into the preceding sentence.
- Reference [4] (a sub-Saharan Africa–specific analysis) is now signaled as regional evidence and paired with an LMIC-wide source supporting the general claim.
- A sentence has been added stating that “low-resource settings” as used in this review includes underserved populations within high-income countries, consistent with the Inclusion criteria and with the revised search strategy.

2. Intro: The introduction still does not define HCD or discuss it in relation to other defined concepts like UCD or co-creation (Line 172). The justification for the focus on UCD and co-creation is also not very strong – why have UCD and co-creation been chosen as the focus, but not HCD or any other participatory methods? The framing needs to be very clear – if all of these methods are included in the review, then more elaboration is needed on the group of methods as a whole, rather than only highlighting two approaches. You could consider replacing “UCD and co-creation” throughout the paper with “UCD and related methodologies” or similar.

We appreciate the Reviewer for pressing us on this point, which has led to a clearer and more defensible framing of the review. Our intention was never to exclude HCD, co-design, participatory design, or design thinking: our search strategy already contains terms for each of these, and our operational definition of eligibility turns on the user-involvement activities a study describes rather than on the label it applies. The framing in the Introduction did not reflect this. The Reviewer is correct that HCD was invoked without definition and that our emphasis on two named approaches was inconsistent with an eligibility criterion that is, in practice, method-agnostic.

We have addressed the comment in three ways. First, we have added a paragraph to the Introduction defining the family of participatory and user-involving design methodologies UCD, HCD, co-creation, co-design, participatory design, and design thinking setting out what they share (iterative development grounded in users’ contexts) and where they differ (principally in the depth of user involvement, from consultation through to equitable partnership), supported by a new table of working definitions. Second, we have adopted the umbrella phrasing the Reviewer suggests, using “UCD and related participatory design methodologies” as the framing term and reserving specific labels for places where a specific approach is meant. Third, we have made explicit that we do not treat these labels as reliable markers of practice: because the terminology is applied inconsistently, eligibility decisions rest on the activities a study describes, and the author-applied label is recorded as an extraction variable so that terminological variation becomes a finding of the review rather than a filter upon it. Specifically;

- Introduction: the three-sentence terminology passage replaced with a full definitional paragraph covering UCD, HCD, participatory design, co-design, co-creation, and design thinking, and the continuum of user involvement that distinguishes them. HCD is now defined at first use.
- New Table 1 added (“Working definitions of user-involving design approaches”), with columns for term, working definition, distinguishing emphasis, and key source; the search strategy table renumbered accordingly.
- Umbrella phrasing adopted throughout the title, Abstract, Rationale, Objectives, review questions, Inclusion criteria, Data extraction, Data synthesis, and Strengths and limitations.
- Rationale: justification for covering the family strengthened. Added text reads:

“We include the full family of user-involving design approaches rather than a single named method because the terminology is used inconsistently across this literature: studies applying substantively similar methods describe them variously, and studies invoking the same label may describe very different depths of user involvement. Restricting the review to one or two labels would therefore fragment a body of evidence that is methodologically continuous, and would systematically miss relevant work.”

- Inclusion criteria: a sentence added drawing the outer boundary of the family — approaches applied to the design or development of a digital health innovation are eligible; participatory approaches applied solely to service delivery or research governance, with no associated technology, are not.
- Appendix II, Section D: “Stated Methodology/Framework” converted from free text to a coded list (UCD / HCD / co-design / co-creation / participatory design / design thinking / CBPR / other / none stated), with a parallel free-text field capturing the authors’ verbatim wording.

3. Line 166: there is a typo where what should be one sentence has been broken into multiple sentences.

We appreciate that you pointed this out, such errors have the potential to confuse the reader and dampen the message. We have corrected this as appropriate.

4. Line 191: questions 1 and 2 do not mention UCD, though this was one of the main methodologies identified in the Intro. Again, it would be helpful to define a specific focus (I would advocate for including all participatory approaches together for a scoping review of this nature), then to be sure it is consistently applied across the research questions, search methods, etc.

We thank the Reviewer for this observation and for the accompanying recommendation, which we have adopted. Indeed, our review questions were not fully in line with the Introduction: questions 1 and 2 referred only to co-creation, question 3 to “UCD and co-creation”, while the eligibility criteria and search strategy were in fact broader than both.

Following the Reviewer’s advice, and consistent with our response to Comment 2, we have adopted the full family of participatory and user-involving design approaches as the explicit scope of the review and have rewritten the review questions so that this scope is applied uniformly. We have additionally conducted a consistency audit across the title, Abstract, Objectives, review questions, Inclusion criteria, search strategy, and data extraction instrument to confirm that a single scope statement is now used throughout, and have aligned the description of innovation types across all three questions, which was also previously inconsistent. Specifically:

- Review questions reworded. The revised questions read:
- *What participatory and user-involving design approaches e.g. UCD, HCD, co-design, participatory design, and co-creation and which named frameworks, processes, or models (e.g. the Double Diamond, IDEAS, the ISO 9241-210 cycle) are applied in the development of digital health innovations for low-resource settings, and how does their use vary across regions, healthcare contexts, and technology types?*
- *How are participatory and user-involving design processes reported to influence the design and functionality of digital health innovations in low-resource settings — at which stages of the development lifecycle, using which methods, with which stakeholder groups, and at what depth of user involvement?*
- *What outcomes, intended and unintended (e.g. usability, acceptability, adoption, clinical accuracy, scalability, sustainability), are reported in association with these approaches in digital health innovations in low-resource settings, and how are these outcomes defined, measured, and reported?*

******A further sub-question has been added in response to Comment 5: How completely are participatory design processes reported in this literature, and what proportion of studies invoke a named approach without describing the activities undertaken?

- Objectives section and Abstract (Objective and Inclusion criteria) revised to carry the same scope statement.
- Consistency audit completed across all sections naming the scope; the eligibility paragraph under “Types of studies”, which was already method-agnostic, is unchanged in substance.
- Formatting corrected: the review questions and the Inclusion criteria headings had been formatted as a single continuous numbered list and are now correctly structured.

5. Line 203: couldn’t “diagnostic innovations” already fit under the umbrella of digital health? It feels like you are mentioning a sub-area of digital health somewhat randomly, and only in some places of the protocol. This also applies to the title.

We thank the Reviewer for bringing this up and respectfully push back on the point. Digital diagnostics are indeed a sub-category of digital health, and our paired phrasing “digital health and diagnostics” only appeared redundant (not all diagnostic technologies have a digital component) however, our scope is limited to diagnostic innovations with a digital component for example smartphone-based microscopy, AI-assisted image analysis, connected point-of-care readers, and digitally enabled diagnostic decision support all of which fall within the definition of digital health we adopt. Purely analogue diagnostic tests without a digital component are not eligible.

We have therefore removed the paired construction throughout and now refer consistently to digital health innovations, and we have amended the title accordingly. To preserve transparency without reintroducing redundancy, we retain digital diagnostics as an explicitly named category within our taxonomy of eligible digital health interventions, and we state once, in the Rationale, that the review pays particular attention to diagnostic applications given the persistent diagnostic gap in low-resource settings and the framing of the wider DI-DIDA project. Innovation type is recorded at extraction, so the distribution of diagnostic and other digital health applications will be reported as a finding rather than asserted as a scope boundary. Specifically

- Title revised to remove “and diagnostics”; the revised title reads: “User-centred design and related participatory approaches in digital health innovation for low-resource settings: a scoping review protocol”.
- Paired phrasing removed from the Abstract (Objective and Methods), review question 3, the Data extraction section, and Strengths and limitations.
- Abstract, Introduction: the sentence narrowing the evidence gap to diagnostics rewritten to describe the gap across digital health innovation as a whole.
- Inclusion criteria, Context: the intervention list now names “digitally enabled diagnostics and point-of-care testing” alongside eHealth, mHealth, telehealth, and wearables, and an explicit exclusion has been added:
  i. Diagnostic tests without a digital component (e.g. conventional lateral-flow rapid diagnostic tests read by eye) are not eligible unless the innovation includes a digital element such as automated reading, image capture and analysis, connectivity, or data capture.
  ii. Rationale: one sentence added stating the review’s particular attention to diagnostic applications and the reason for it.
- Appendix II, Section C retitled “Digital Health Innovation (details)”; technology-type options reviewed to confirm each is unambiguously digital.
- Search strategy: the diagnostic terms are retained without a digital qualifier to preserve sensitivity; the Study selection section now states that the digital-component requirement is applied at screening.

6. Line 210: From “Particularly…” onwards - you do not need to talk about these aspects of the inclusion criteria in the “population/setting” section. They fit better in later sections.

This is a keen observation and important in the logical flow of the paper, thank you for highlighting it. It has been implemented.

7. Line 233: I would argue that including papers that explicitly state they have used these methods but without describing the relevant activities should be included, given your point in the introduction about this being a problem in the field. This seems that it would then be a finding of your review if some proportion of the papers mention UCD/HCD/co-design, etc. but do not explicitly describe their methods.

We thank the Reviewer for this insight, which we found persuasive. The Reviewer is correct that our exclusion rule was in tension with our own framing: having argued that inconsistent and superficial use of participatory design terminology is a recognized problem in this field, it was inconsistent to then remove from the review precisely those studies that exemplify it. We have revised the eligibility criteria so that studies claiming the use of a named participatory or user-involving design approach are eligible whether they describe the underlying activities.

A study is now eligible if it reports the development, adaptation, or evaluation of a digital health innovation in a low-resource setting and either (a) describes at least one concrete user-involvement activity, or (b) explicitly claims the use of a named user-involving design approach without describing the activities undertaken. We have added an extraction variable classifying each included study by the completeness of its reporting fully described, partially described, or label-only and will report this distribution as a primary descriptive finding, disaggregated by publication year, publication type, and region, and by the specific label invoked.

Label-only studies will be analyzed as a distinct stratum rather than pooled with fully described studies, since they cannot contribute data on methods, stages of involvement, or depth of user engagement. Thank you for highlighting this, as this change converts an exclusion into what we expect will be one of the more informative outputs of the review.

- Inclusion criteria, Types of studies: eligibility rule rewritten. The revised text reads:

To be eligible, a study must report the development, adaptation, or evaluation of a digital health innovation in a low-resource setting, and must meet at least one of the following: (a) it describes at least one concrete user-involvement activity (e.g. interviews, focus groups, contextual inquiry, participatory prototyping, co-design workshops, or usability testing) with end-users or other relevant stakeholders at one or more stages of development; or (b) it explicitly claims the use of a named user-involving design approach including UCD, HCD, co-design, co-creation, participatory design, or design thinking without describing the associated activities. Studies meeting only criterion (b) are retained deliberately: the inconsistent and sometimes nominal use of this terminology is itself a recognized feature of the field, and quantifying its extent is an objective of this review.

- A sub-question added to the review questions and mirrored in the Objectives and Abstract.
- Appendix II, Section D: new field “Reporting completeness” added (fully described / partially described / label-only), with a decision rule based on whether specific methods, participant groups, and development stages are identifiable. The rule will be piloted during calibration of the extraction form.
- Data synthesis: reporting completeness described and cross-tabulated by year, region, publication type, and stated approach; label-only studies excluded from the synthesis of methods, stages, and depth of involvement and reported separately.

8. Line 242: Search should not stop at 2025, but should go until the present

We agree with this sentiment and have implemented it as such

9. Line 251: reference needed for JBI

We are grateful that you were able to pick up on this, proper reference to the reporting framework is important.

10. Line 260: do you mean “peer-reviewed literature” or “academic literature” and “grey literature” instead of “published” and “unpublished”?

This is a telling observation for the phrasing. What we mean here included published peer reviewed academic and grey literature. We agree that a proper clarification is to be made.

11. Line 276: I don’t think #6 should be included in your search – this could exclude relevant papers that refer to high-resource settings, or papers in high-income countries that focus on low-resource communities or populations. Similarly, if you want to include papers focused on underserved communities in high-income settings, as stated in your Methods, you should include search terms related to these populations (e.g., underserved, low-income, marginalized, etc.)

We are grateful to the Reviewer for this correction, which identified a genuine inconsistency between our search strategy and our stated eligibility criteria. The NOT block (#6/#7) was intended to improve precision but would have done so at an unacceptable cost to sensitivity: it would have removed any record merely mentioning a high-income comparator and, more seriously, would have systematically excluded the studies we state we wish to include those conducted with underserved or marginalized populations within high-income countries. We have removed the NOT block entirely from all database searches.

Following the Reviewer’s second point, we have added a population facet capturing studies of underserved populations irrespective of national income classification, using terms including underserved, marginalized, disadvantaged, low-income, deprived, vulnerable, resource-limited, resource-constrained, rural, remote, refugee, migrant, Indigenous, and informal settlement or slum. This facet is combined with the geographic facet using OR, and the result combined with the design-methods and digital health facets using AND. We have also replaced our hand-constructed country list with the Cochrane EPOC low- and middle-income country search filter, adapted for each database, providing a validated and citable basis for the geographic facet.

We note that Reviewer #2 (Comment 2) raised a related concern from the opposite direction, questioning whether underserved populations in high-income countries should be eligible at all, and asking for explicit criteria if they are. Our resolution applies both recommendations at different stages of the process: the search is deliberately sensitive, as this Reviewer asks, while eligibility is determined at screening by a strict, externally anchored definition of a low-resource setting, as Reviewer #2 asks. The full definition is set out in our response to Reviewer #2, Comment 2 and is reproduced in the Inclusion criteria.

- Table 1: rows #6 and #7 (the NOT block) deleted; facets renumbered. The revised structure is #1 design methods, #2 digital health, #3 geographic (Cochrane EPOC LMIC filter), #4 underserved populations, #5 = #3 OR #4, #6 = #1 AND #2 AND #5.
- New population facet added, comprising controlled vocabulary (Vulnerable Populations, Medically Underserved Area, Poverty Areas, Rural Population, Transients and Migrants, Refugees, Indigenous Peoples) and the free-text terms listed above.
- Inclusion criteria, Population/setting: strict operational definition added (see response to Reviewer #2, Comment 2), applied at screening rather than in the search string.
- Study selection: setting eligibility among the criteria on which the two independent reviewers will be calibrated in a pilot screening round.
- Appendix I now presents the complete, untruncated search string for every database, with the date run and the number of records retrieved, in accordance with PRISMA-S.
- Study status and timeline updated to reflect the substantially larger yield expected from the revised strategy.

12. Line 305: the editor can make the final call here, but I don’t think you need to mention these ethics approvals – they are not relevant for this scoping review and just take up unnecessary space, in my opinion.

We agree with this sentiment and have implemented it as such

13. Line 318: it appears that this search has already been conducted, but this is concerning as I have identified issues with the search terms that I think need to be corrected before the scoping review is completed.

We appreciate the Reviewer raising this and we take the concern seriously. By way of clarification, and as stated in the Study status and timeline section, this protocol describes a review that is in progress: at the time of original submission the initial search had been executed. We recognize that publishing a protocol for a review already underway carries an obligation of transparency, and we accept the Reviewer’s implicit point that a protocol serves little purpose if the concerns its surfaces cannot still change the conduct of the review.

We wish to be unambiguous on the substantive question: we will re-run the search in full using the corrected strategy before completing the review. The corrections arising from this review process removal of the NOT block excluding high-income settings and addition of the underserved-population facet (Comment 6), adoption of the validated Cochrane EPOC LMIC filter (Comment 6), inclusion of the wider family of participatory design terms (Comments 2 and 3), and revised handling of diagnostic terminology (Comment 4) — materially change what the search retrieves, and we agree they must be applied before rather than after completion. Records retrieved by the original search are retained, and all records will be screened against the revised eligibility criteria; no eligibility decision made under the superseded criteria will be carried forward without re-screening.

We have revised the Study status and timeline section to report the current stage accurately, to give the date of the original search and the planned date of the corrected search, and to commit to reporting both in the PRISMA-ScR flow diagram of the completed review. We have also added an amendments log recording each change to the search strategy and eligibility criteria arising from peer review, with its rationale, so that the provenance of the final strategy is fully traceable. Specifically:

- Study status and timeline rewritten to state the date of the original search, the databases searched, the number of records retrieved, the current screening status, and the planned date of the corrected search. The superseded projected dates have been removed.
- New “Protocol amendments” subsection added, presenting a dated table of each amendment (section, change, rationale) arising from peer review.
- The protocol has been registered on the Open Science Framework [registration DOI and date to be inserted]; the registration is now cited in the Methods.
- Appendix I presents both the original and the corrected search strings, labelled by date.
- Methods: both searches will be reported as distinct streams in the PRISMA-ScR flow diagram of the completed review.

14. Line 493: You should state in the protocol where the list of LMICs came from (e.g. World Bank classification and year) – this is currently only in your response to my comment, but not in the Methods.

We thank the Reviewer for catching this omission. The Reviewer is right that a clarification offered in a response letter does not benefit readers of the published protocol, and we have moved this information into the manuscript itself.

The Methods now state that eligible countries were identified using the World Bank income classification for fiscal year [YYYY], comprising economies classified as low-income, lower-middle-income, or upper-middle-income, with the full list and its source reproduced in Appendix I alongside the search strings. Consistent with our response to Comment 6, the geographic facet of the search now adapts the Cochrane EPOC low- and middle-income country search filter, which is itself derived from the World Bank classification; the filter, its version, and the date of access are cited. We have also specified how countries whose classification changed during the 2015–2025 window will be handled.

Prompted by this comment we have reviewed our other responses and moved into the manuscript any clarification that had previously been offered only in correspondence. Specifically:

•	Methods, Search strategy: source of the country list stated. Added text reads:

Countries eligible as low- and middle-income were identified using the World Bank income classification for fiscal year [YYYY] (low-income, lower-middle-income, and upper-middle-income economies). The geographic component of the search adapts the Cochrane EPOC low- and middle-income country search filter [version; date accessed], which is based on this classification. The full country list is reproduced in Appendix I.

- Both the World Bank classification and the Cochrane EPOC filter are now formally cited in the reference list, with access dates.
- Appendix I: the full country list reproduced as a table, with the classification year given in the caption.
- Inclusion criteria: reclassification rule added — eligibility is determined by the country’s classification at the time the study was conducted; where the year of conduct cannot be determined, the classification at year of publication is applied and recorded at extraction.
- Appendix II, Section B: a field recording the classification year applied has been added; the WHO region field has been corrected to include the European Region, which was omitted.
- All clarifications given in this response letter have been checked against the manuscript to confirm each has a corresponding revision in the text.

15. Line 271: “In several electronic databases” is vague - it is still not very clear which databases will be searched. It is also not yet clear how grey literature will be located, as “grey literature sources” is quite vague too.

We thank the Reviewer for this comment, which prompted us to review the Methods carefully. We had initially named three different sets of databases in three places the Abstract (PubMed, Scopus, IEEE Xplore, Web of Science), the Search strategy section (MEDLINE and CINAHL for the initial limited search, then “several electronic databases” together with Web of Science and Google Scholar), and the Study selection section (MEDLINE, Embase, Scopus, Web of Science, Google Scholar, IEEE Xplore). This was an error and we are grateful it was identified. We have replaced all three with a single definitive list, applied consistently, and presented as a table specifying for each source the host platform, the coverage searched, and the rationale for inclusion. We have added the ACM Digital Library, which indexes the CHI, DIS, COMPASS, and ICTD proceedings where a substantial share of participatory design research with LMIC populations appears, and the WHO Global Index Medicus, which indexes regional journals from the settings this review targets.

On grey literature we accept that our description was insufficiently specific to be reproducible. We have replaced the general reference to “grey literature sources” with a named list of repositories and organizational websites, together with the search approach for each, and now state that grey literature searching follows the same date limits and eligibility criteria as the database searches, will be conducted by a named reviewer, and will be documented with the date searched and the number of records retrieved from each source. Specifically:

- The three inconsistent database lists reconciled into one definitive list, applied in the Abstract, the Search strategy section, and the Study selection section.
- New table added specifying, for each database: MEDLINE (PubMed), Embase (Elsevier), CINAHL (EBSCOhost), Scopus, Web of Science Core Collection, IEEE Xplore, ACM Digital Library, WHO Global Index Medicus, and Google Scholar (first 200 results screened), together with platform, coverage searched, and rationale.
- New table added specifying grey literature sources and approach: preprint servers (medRxiv, arXiv cs.HC); theses and dissertations (ProQuest Dissertations & Theses Global; institutional repositories via BASE and CORE); organizational repositories (WHO IRIS, World Bank Open Knowledge Repository, USAID Development Experience Clearinghouse, UNICEF); organizational websites (PATH, FIND, Digital Square, Global Digital Health Network); and targeted web searching (first 100 Google results per string, logged with date).
- Eligibility boundary for grey literature stated, including treatment of preprints where a peer-reviewed version also exists, and exclusion of commercial product documentation.
- The three-step search description revised to the past tense for the initial limited search, which has been completed and informed the strategy presented.
- Remaining PRISMA-S items added: dates searched, records retrieved per source, involvement of an information specialist and whether the strategy underwent PRESS peer review, duplicate-handling procedure and software, and all limits applied.

16. Appendix: you are missing search terms with the British spelling of “human-centred” and “user-centred”

We agree with this observation and have corrected it as such

Reviewer #2: - The introduction is well structured and clearly motivates the review. However, some technology-specific examples (e.g., malaria diagnostics, wearable monitoring, and point-of-care technologies) are described in greater detail than is necessary for a protocol. As the review focuses on UCD and co-creation rather than digital health technologies themselves, these examples could be condensed to place greater emphasis on the conceptual rationale and the implementation challenges motivating the review.

**Response**

We thank the Reviewer for this observation and for the encouraging assessment of the Introduction’s structure. We agree that the technology-specific passages were disproportionately detailed for a protocol whose subject is design methodology rather than the technologies themselves. On re-reading, we found that four consecutive paragraphs covering digital diagnostics for malaria and schistosomiasis, clinical decision support for antimicrobial resistance, point-of-care testing, and wearable monitoring together accounted for approximately 359 of roughly 1,013 words in the Introduction, while contributing relatively little to the argument for why user-centered design matters. The passage on wearables in particular described photoplethysmography, motion-artifact cancellation, pulse transit time, and respiratory sinus arrhythmia at a level of technical granularity not relevant to the review’s aims.

We have condensed these four paragraphs into two, retaining a small number of illustrative examples to establish that digital health encompasses a diverse set of technologies relevant to low-resource settings, but removing the device-level technical description. The space recovered has been redirected to the conceptual rationale, which we have expanded in two respects: a fuller account of the design methodologies under study and how they relate to one another, and a stronger statement of the implementation challenge the gap between technical performance and real-world adoption that motivates the review. This has allowed us to address Reviewer #1’s request for definitions of HCD and related approaches without lengthening the Introduction overall.

**Key changes made**

- Introduction paragraphs 4–7 condensed into two (≈150 words in place of ≈359). Revised text reads:

Digital health innovations span a wide range of applications relevant to low-resource settings. These include diagnostic tools such as smartphone-based microscopy and AI-assisted image analysis for malaria and schistosomiasis [10,11]; clinical decision support systems that assist providers in identifying resistant organisms and selecting appropriate treatment, with implications for antimicrobial stewardship [12]; digitally enabled point-of-care testing, which may help close diagnostic service gaps in underserved and remote areas [13]; and wearable sensors supporting continuous monitoring of chronic conditions, allowing a shift from episodic clinic-based measurement towards remote monitoring and risk-based triage [17–22].

What these applications share is that their potential is contingent on use. Technical performance established under controlled conditions does not by itself determine whether a tool is adopted, used correctly, or sustained in the settings where it is deployed — and it is this gap between demonstrated capability and realized benefit that motivates attention to how such technologies are designed.

- Device-level technical description deleted, including the passage on multi-wavelength photoplethysmography, motion-artifact cancellation, and personalized baseline calibration.
- Telemedicine and mHealth examples in paragraph 3 trimmed.
- Space recovered redirected to the definitional paragraph on design methodologies (Reviewer #1, Comment 2) and to a strengthened statement of the implementation challenge, drawing on Greenhalgh et al. [23].
- References reviewed in consequence: grouped citation adopted for the condensed passages, and reference [9] (a LinkedIn post) replaced with a peer-reviewed source.

The operational definition of “low-resource settings” requires further clarification. The eligibility criteria currently include LMICs as well as rural or underserved areas in high-income countries, but it is not clear why the latter are included or how they will be identified during screening. Rurality or underserved status does not necessarily equate to a low-resource setting. The authors should either (i) adopt a standard definition (e.g., World Bank LMIC classification), or (ii) provide a clear conceptual and operational justification for extending the review to resource-constrained populations within high-income countries, including explicit eligibility criteria.

We appreciate this comment, which identifies a genuine weakness. We accept the central point: rurality or underserved status does not in itself constitute a low-resource setting, and our criteria as written would have permitted inclusion based on an author’s descriptive label rather than any assessable characteristic of the setting.

We note that Reviewer #1 raised a related but opposite concern (Comment 6), observing that our search strategy would have excluded studies of underserved communities within high-income countries despite our eligibility criteria admitting them. Taken together, the two comments identify the same underlying problem from different directions: we had extended eligibility to resource-constrained populations in high-income countries without either justifying the extension conceptually or specifying how it would be applied. We have chosen option (ii) retaining the extension, but supplying both the justification and explicit, assessable criteria.

As a justification the construct that matters for this review is *resource constraint* as it bears on the design and deployment of digital health innovations: intermittent connectivity and infrastructure limitations, workforce shortage, constrained financing, heterogeneous digital and health literacy, linguistic diversity, and structural barriers to access. These conditions are concentrated in low- and middle-income countries but are not confined to them, and where they obtain within a high-income country whether in remote and rural communities, informal settlements, refugee and undocumented migrant populations, and indigenous communities served by under-resourced systems the design problem confronting a development team is substantively the same. Because our review question concerns how design methods are adapted to such conditions, restricting eligibility to national income classification alone would exclude directly informative evidence based on a unit of analysis (the country) that does not match the unit at which the constraint operates (the setting and population). We recognize, equally, that this reasoning does not license inclusion of any population described as rural or disadvantaged, and our revised criteria are correspondingly narrow.

Therefore, a study conducted in a high-income country is eligible only if both of the following are satisfied: (a) the study explicitly frames the innovation as addressing resource constraints or structural barriers to healthcare access for the target population, rather than merely reporting that participants were rural or of low income; and (b) the population meets at least one externally defined criterion drawn from a specified list of national or international designations. Meeting (b) alone is not sufficient. The specific criterion satisfied is recorded during extraction, so that the basis for every such inclusion is auditable.

We have additionally specified that studies from high-income settings will be reported separately in the synthesis, and that we will present our principal findings both including and excluding them, so that readers can assess whether the extension materially affects the review’s conclusions.

**Key changes**

- Inclusion criteria, Population/setting: definition replaced. The revised text reads:

For the purposes of this review, a low-resource setting is defined as one in which the design and deployment of digital health innovations is materially constrained by limitations in infrastructure, health workforce, financing, connectivity, or population-level digital and health literacy, or by structural barriers to healthcare access. Studies are eligible if conducted in (i) a country classified by the World Bank as low-income, lower-middle-income, or upper-middle-income at the time the study was conducted; or (ii) a defined population or geographic area within a high-income country, where both of the following apply: (a) the study explicitly frames the innovation as responding to resource constraints or structural barriers to access affecting that population; and (b) the population corresponds to at least one externally defined designation of underservice or deprivation.

- Appendix I: list of accepted external designations added, with citations — health workforce and service shortage designations (e.g. HRSA Medically Underserved Area or Population; Health Professional Shortage Area); rural and remote classifications (e.g. RUCA codes, Eurostat DEGURBA, Modified Monash Model); area deprivation indices (most deprived quintile of a recognized national index); population-group designations (refugees, asylum seekers, undocumented migrants, Indigenous communities served by under-resourced systems, people experiencing homelessness, incarcerated populations); and informal settlements meeting UN-Habitat criteria.
- Appendix II, Section B: new fields added — setting eligibility route (LMIC / high-income underserved), designation met, and the verbatim resource-constraint rationale stated by the authors.
- Data synthesis: findings reported separately for LMIC and high-income underserved studies, with principal descriptive results presented both with and without the latter.
- Study selection: setting eligibility included among the criteria for pilot calibration of the two independent reviewers, with inter-rater agreement reported for this criterion.
- Strengths and limitations: the judgement involved in this boundary acknowledged, together with the mitigations above.

The “Study status and timeline” section appears to contain projected dates that have now passed. Please update this section to reflect the status of the review (e.g., whether the literature search, screening, or data extraction have been completed). If the review has progressed since the protocol was first submitted, it would be helpful to clarify that the protocol continues to reflect the methods that were followed, and that any deviations will be reported in the final review.

We thank the Reviewer for this comment, which Reviewer #1 also raised from a different angle (Comment 7), and we have addressed both together. The Reviewer is correct that the projected dates had been overtaken by events, and we apologize for the oversight.

We have rewritten the Study status and timeline section to report the position accurately: the date on which the initial search was executed, the databases searched and records retrieved, the current stage of screening and extraction, and revised projected dates for the remaining stages. We have also added the clarification the Reviewer suggests and made it more specific in one respect. Because the peer review of this protocol has itself prompted substantive changes to the search strategy and eligibility criteria principally the removal of the exclusion of high-income settings, the addition of a population facet for underserved communities, the broadening of the design-methodology terms, and the revised treatment of studies that name a design approach without describing it the protocol as originally submitted does not fully describe the methods that will now be followed. We have therefore committed to re-running the search under the corrected strategy and to re-screening all records against the revised criteria, so that the revised protocol, rather than the original, is the document the completed review follows.

To make this traceable, we have added a protocol amendments log recording each change, its date, and its rationale. The Study status section now states that the review will be conducted in accordance with this revised protocol, that both the original and corrected searches will be reported in the PRISMA-ScR flow diagram, and that any subsequent deviation from the revised protocol will be reported explicitly in the final review, with its rationale and an assessment of its likely effect on the findings.

**Key changes**

- Study status and timeline rewritten; all superseded projected dates replaced.
- Protocol fidelity statement added:

This review will be conducted in accordance with the methods described in this revised protocol. Amendments made to the protocol following peer review are recorded, with dates and rationale, in [Protocol amendments]. Any deviation from the revised protocol occurring during the conduct of the review will be reported in the final publication, together with its rationale and an assessment of its likely effect on the findings.

- Revised timeline allows for the increased screening yield arising from the broadened eligibility criteria and search strategy.
- Tense corrected throughout the Methods and Abstract so that completed steps are described in the past tense.

RQ2 asks: “How do co-creation processes influence…”. But, a scoping review maps reported evidence rather than demonstrating causal influence. A more cautious wording could be: “How are co-creation processes reported to influence…”.

We thank the Reviewer for this correction, which we accept without reservation. The original wording implied a causal claim that a scoping review is not designed to support we can map what studies report about the relationship between design processes and their outcomes, but we cannot establish influence. We have adopted the Reviewer’s suggested phrasing.

Reviewer #1 raised a related concern about the consistency of the review questions (Comment 3), and we have taken the opportunity to revise all three together. Review question 2 now reads: “How are participatory and user-involving design processes reported to influence the design and functionality of digital health innovations in low-resource settings — at which stages of the development lifecycle, using which methods, with which stakeholder groups, and at what depth of user involvement?” Question 3 has likewise been adjusted so that outcomes are described as reported in association with these approaches rather than as arising from them.

Prompted by this comment, we have also reviewed the Objectives, Abstract, and Data synthesis sections for similar over-claiming, and have amended several instances in which the language of mapping had slipped into the language of effect. We have added a sentence to the Data synthesis section stating explicitly that the review characterizes what is reported, and that associations between design processes and outcomes are presented as reported by study authors rather than as findings of the review.

**Key changes**

- Review question 2 revised using the Reviewer’s suggested wording; review question 3 confirmed as “reported in association with”.
- Causal language amended in the Introduction for example “enhances usability but also increases user satisfaction” recast as “has been reported to enhance… and to increase…”, and “can lead to more personalized and impactful healthcare solutions” recast as “have been described as contributing to”.
- Data synthesis: framing sentence added:

Throughout, this review characterizes what studies report. Where relationships between design processes and outcomes are described, these are presented as reported by the original authors; the review does not assess the validity of these claims or establish causal relationships between design approach and outcome.

- Strengths and limitations: a sentence added noting that reported associations between design process and outcome are typically uncontrolled and frequently reported by the design teams themselves, which constrains what can be inferred.

Limitations may also acknowledge language restriction (English/French), and the anticipated heterogeneity of interventions, co-creation approaches, and reported outcomes, which may limit the comparability of findings.

We agree that the language restriction warrants explicit acknowledgement, particularly given the subject of this review: restricting to English and French will disproportionately exclude literature from Latin America, Lusophone Africa, China, and Arabic-speaking countries that is, from precisely the low-resource settings the review seeks to characterize and therefore carries a risk of geographic bias that is not incidental to our aims but bears directly on them. The restriction is pragmatic, reflecting the language capabilities of the review team and the resources available for translation, and we now state this openly rather than presenting it as a methodological choice. As a partial mitigation we have added the WHO Global Index Medicus to our sources, which indexes regional databases including LILACS with English-language abstracts; and we will record and report the number of records excluded on language grounds, by language, so that readers can gauge the potential extent of the omission.

On heterogeneity, we agree and have expanded the limitations accordingly. We anticipate substantial variation along at least four dimensions: the technologies studied; the design approaches applied and the depth of user involvement they entail; the outcomes reported and the instruments used to measure them, few of which are standardized; and the settings themselves. This variation is expected; it is in part what the review sets out to map, but it limits comparability across studies and precludes any pooling or aggregate statement about effectiveness. We have made it explicit that the review’s contribution is descriptive. We have also noted that, consistent with JBI guidance for scoping reviews, we do not undertake critical appraisal of included studies, so no assessment of the reliability of reported outcomes is offered.

Prompted by this comment we have restructured the Strengths and limitations section, which had been a single undifferentiated paragraph, into clearly separated statements of strengths and limitations.

Key changes

- Strengths and limitations restructured into separated statements of strengths and of limitations.
- Language limitation added:
- This review is restricted to literature published in English and French, reflecting the language capabilities of the review team. This restriction risks under-representing evidence from Spanish- and Portuguese-speaking Latin America, Lusophone Africa, and Arabic- and Chinese-language literature, and therefore introduces a potential geographic bias that is material given the review’s focus on low-resource settings. We partially mitigate this through the inclusion of the WHO Global Index Medicus, and we will record and report the number of records excluded on language grounds, disaggregated by language.
- Heterogeneity limitation added, covering technologies, design approaches and depth of involvement, outcomes and measurement instruments, and settings, and stating that the review’s contribution is descriptive rather than comparative.
- Absence of critical appraisal acknowledged, consistent with JBI guidance for scoping reviews.
- Limitation on the reproducibility of grey literature and Google Scholar searching added.
- The existing limitation on terminological inconsistency rewritten, since studies using labels loosely are now eligible by design (Reviewer #1, Comment 5); the residual limitation is the risk of omitting studies that use participatory methods without naming them.
- Screening procedure and PRISMA-ScR flow diagram amended so that language exclusions are counted and reported rather than discarded silently.

Reviewer #3: I have carefully examined both the authors' response to the previous reviewer and the revised manuscript. The authors have made substantial efforts to address the concerns raised, and the manuscript has improved considerably. In particular, the scope of the review is now more clearly defined, the rationale is better aligned with the stated objectives, the review questions are more appropriate for a scoping review methodology, and the methodological description is more transparent and reproducible. The revised protocol is generally well organized and follows accepted guidance for scoping reviews. Overall, these revisions adequately address the substantive concerns raised by the previous reviewer. I only have a small number of minor observations before acceptance.

We appreciate the reviewer for taking the time to contribute to this work.

1. The Introduction remains somewhat lengthy- Although the broader examples of digital health have improved the manuscript, several sections describing individual technologies (for example wearable technologies, disease-specific examples and technical descriptions) appear more detailed than is necessary for a protocol. Some condensation would improve readability without affecting the scientific contribution. This is an editorial suggestion rather than a methodological concern.

We thank the Reviewer for this suggestion and note that Reviewer #2 made a closely related observation (Comment 1). Two Reviewers independently identifying the same imbalance gave us confidence to condense more decisively than we might otherwise have done, and we have made a single set of edits addressing both comments.

On examination, four consecutive paragraphs describing disease-specific diagnostic examples, point-of-care testing, and wearable monitoring accounted for approximately 359 of the Introduction’s roughly 1,013 words. The passage on wearables was the most technically detailed, describing multi-wavelength photoplethysmography, motion-artifact cancellation, pulse transit time, and respiratory sinus arrhythmia — a level of granularity that serves no purpose in a protocol concerned with design methodology. We have condensed these four paragraphs into two.

We have been careful, in doing so, to distinguish breadth from depth. The Reviewer notes that the broader range of digital health examples improved the manuscript, and we have retained that breadth: the revised text still spans diagnostics, decision support, point-of-care testing, telemedicine, mHealth, and wearables, so that the diversity of technologies within scope remains evident. What we have removed is the technical description of how individual devices function. The Introduction is now approximately [X] words, and the space recovered has been redirected to the conceptual rationale — specifically to the definitions of the design methodologies requested by Reviewer #1 (Comment 2) and to a fuller statement of the implementation challenge motivating the review.

Key changes

- Addressed by the same edits described in our response to Reviewer #2, Comment 1.
- Technology categories retained in full — diagnostics, clinical decision support, point-of-care testing, telemedicine, mHealth, and wearables — so that the breadth the Reviewer credits as an improvement is preserved; only device-level technical description has been removed.
- Revised Introduction word count inserted in the text and reported identically in both responses.

2. The rationale could be slightly more concise- While the rationale is now much stronger, there remains some repetition around the value of documenting UCD/co-creation and the need for evidence mapping. Streamlining this section would improve flow.

We thank the Reviewer for both the encouragement and the observation. On re-reading the section we found the repetition to be more pronounced than we had appreciated: of six paragraphs totaling 388 words, four consecutive paragraphs each made a version of the same argument that the absence of systematic documentation impedes the identification of good practice, the replication of successful approaches, and the scaling of initiatives, and that better documentation would therefore improve relevance, sustainability, and health outcomes. The point is a sound one but did not require four statements.

We have restructured the section around a single line of argument: low-resource settings present distinctive conditions that shape whether digital health innovations are adopted and sustained; participatory design approaches are well suited to those conditions; what is unclear is not whether they can be applied but how they are applied, with which methods, at which stages, with which stakeholders, and to what reported effect; and that evidence is currently scattered and unmapped, which prevents the identification of transferable practice. The four repetitive paragraphs have been consolidated into one, and the section reduced from 388 to approximately [X] words without the loss of any distinct claim.

We have made two further changes prompted by this comment. First, we have removed the definitional aside in the opening paragraph contrasting UCD and co-creation, which now duplicates the fuller treatment added to the Introduction in response to Reviewer #1 (Comment 2). Second, we have relocated the material on establishing frameworks and enhancing sustainability, which described desirable future developments rather than the rationale for this review, to the discussion of the review’s anticipated contribution.

Key changes

- Paragraphs 3–6 consolidated into a single paragraph (≈55 words in place of ≈182). Revised text reads:
- Without systematic documentation of how these approaches are applied and with what results, it is difficult to identify transferable practice, to replicate approaches that have worked elsewhere, or to judge which elements of a design process account for reported outcomes. Mapping this evidence is a precondition for any of these.
- Paragraph 1: the definitional sentence contrasting UCD and co-creation removed, as it now duplicates the Introduction.
- Paragraph 2 retained substantially intact as the core of the argument; the sentence fragment beginning “Current literature indicates that while there are examples of co-creation in practice…” joined to the following sentence, and the missing punctuation after “distinctive realities” supplied.
- Paragraph 4 relocated to the discussion of the review’s anticipated contribution in Strengths and limitations.
- Net length of the section checked after incorporating the additions arising from Reviewer #1, Comments 2 and 4, to confirm an overall reduction.

3. A final proofread would be beneficial- I noticed occasional grammatical and stylistic inconsistencies (for example punctuation, sentence flow and wording) that would benefit from careful copy-editing prior to publication. These do not affect the scientific content but would improve readability. The authors have responded appropriately and comprehensively to the previous review. The remaining observations are minor editorial suggestions rather than substantive methodological concerns. I therefore recommend acceptance after minor editorial revisions.

RESPONSE

We thank the Reviewer for this comment and accept it without qualification. The manuscript has been fully copy-edited prior to resubmission, and we have engaged professional copy-editing support for the final version. We are grateful to the Reviewer for the courtesy of flagging this separately from the methodological comments.

In addition to a general edit, we have systematically addressed the specific classes of error we identified. These included sentence fragments in the Introduction, the Rationale, and the Methods; inconsistent placement of citations relative to sentence-final punctuation, affecting some forty instances; inconsistent hyphenation and capitalization of key terms; mixed American and British spelling conventions; inconsistent appendix numbering; stray double spaces; and missing terminal punctuation. We have also corrected the use of “low-resourced settings” in the title, adopting “low-resource settings” consistently in line with the usage in the body of the manuscript.

Two substantive typographical errors in the data extraction instrument were identified during this process and corrected: in Appendix II, Section C, “HER/Dashboards” should read “EHR/Dashboards”; and in Section D, “Personal development”, listed among UCD and co-creation methods, should read “Persona development”. We are grateful that this comment prompted a line-by-line review, as these would have propagated into the conduct of the review itself.

Finally, we have corrected the document’s structural formatting. Several items the components of the intervention taxonomy under “Context”, and the Inclusion criteria and Population/setting headings had been formatted as headings or list items in ways that misrepresented the document’s hierarchy, and these have been reset to the correct styles.

In closing

We thank all three Reviewers for their careful and constructive reading. Between them the comments identified several genuine inconsistencies in regional framing, in the scope statement across sections, in the database lists, between the search strategy and the eligibility criteria, and in the reported status of the work together with two substantive errors in the data extraction instrument. The protocol is materially stronger for their correction.

We are particularly grateful for the two comments that changed the design of the review rather than its presentation: Reviewer #1’s argument that studies invoking design terminology without describing it should be included and reported as a finding, and Reviewer #2’s insistence that the extension to underserved populations in high-income countries be justified and made operational. Both have improved the review’s contribution. We hope the revisions are satisfactory and would be glad to provide any further clarification the Reviewers or Editor may require.
