# Supplementary material for: User-centered design and related participatory approaches in digital health innovations for low-resource settings: A scoping review protocol
Source: PLoS One. 2026 Sep 8;21(9):e0337671. doi: 10.1371/journal.pone.0337671 (PMC13552726; doi:10.1371/journal.pone.0337671)
Supplement: Peer review history

# Peer review: Decision Letter 0

**DOI:** 10.1371/journal.pone.0337671.r001

**Authors:** Helen Howard

12 Jun 2026

User-centered design and co-creation for digital health Innovations and diagnostics: A scoping review protocol to explore applications and outcomes in low-resource settings

PLOS One

Dear Dr. Otukpa,

Thank you for submitting your manuscript to PLOS ONE. After careful consideration, we feel that it has merit but does not fully meet PLOS ONE’s publication criteria as it currently stands. Therefore, we invite you to submit a revised version of the manuscript that addresses the points raised during the review process.

Please submit your revised manuscript by Jul 27 2026 11:59PM. If you will need more time than this to complete your revisions, please reply to this message or contact the journal office at plosone@plos.org. When you're ready to submit your revision, log on to https://www.editorialmanager.com/pone/ and select the 'Submissions Needing Revision' folder to locate your manuscript file.

Please include the following items when submitting your revised manuscript:

- A letter that responds to each point raised by the academic editor and reviewer(s). You should upload this letter as a separate file labeled 'Response to Reviewers'.
- A marked-up copy of your manuscript that highlights changes made to the original version. You should upload this as a separate file labeled 'Revised Manuscript with Track Changes'.
- An unmarked version of your revised paper without tracked changes. You should upload this as a separate file labeled 'Manuscript'.

If you would like to make changes to your financial disclosure, please include your updated statement in your cover letter. Guidelines for resubmitting your figure files are available below the reviewer comments at the end of this letter.

If applicable, we recommend that you deposit your laboratory protocols in protocols.io to enhance the reproducibility of your results. Protocols.io assigns your protocol its own identifier (DOI) so that it can be cited independently in the future. For instructions see: https://journals.plos.org/plosone/s/submission-guidelines#loc-laboratory-protocols. Additionally, PLOS ONE offers an option for publishing peer-reviewed Lab Protocol articles, which describe protocols hosted on protocols.io. Read more information on sharing protocols at https://plos.org/protocols?utm_medium=editorial-email&utm_source=authorletters&utm_campaign=protocols.

As the corresponding author, your ORCID iD is verified in the submission system and will appear in the published article. PLOS supports the use of ORCID, and we encourage all coauthors to register for an ORCID iD and use it as well. Please encourage your coauthors to verify their ORCID iD within the submission system before final acceptance, as unverified ORCID iDs will not appear in the published article. *Only*  the individual author can complete the verification step; PLOS staff *cannot*  verify ORCID iDs on behalf of authors.

We look forward to receiving your revised manuscript.

Kind regards,

Helen Howard

Staff Editor

PLOS One

Journal Requirements:

When submitting your revision, we need you to address these additional requirements.

1.Please ensure that your manuscript meets PLOS ONE's style requirements, including those for file naming. The PLOS ONE style templates can be found at

https://journals.plos.org/plosone/s/file?id=wjVg/PLOSOne_formatting_sample_main_body.pdf and

https://journals.plos.org/plosone/s/file?id=ba62/PLOSOne_formatting_sample_title_authors_affiliations.pdf

2. Thank you for stating the following financial disclosure:

“This research is funded by the European Health and Digital Executive Agency (HADEA) Project 101057251 – DI-DIDA”

Please state what role the funders took in the study.  If the funders had no role, please state: "The funders had no role in study design, data collection and analysis, decision to publish, or preparation of the manuscript."

If this statement is not correct you must amend it as needed.

Please include this amended Role of Funder statement in your cover letter; we will change the online submission form on your behalf.

3. Please provide a complete Data Availability Statement in the submission form, ensuring you include all necessary access information or a reason for why you are unable to make your data freely accessible. If your research concerns only data provided within your submission, please write "All data are in the manuscript and/or supporting information files" as your Data Availability Statement.

4. When completing the data availability statement of the submission form, you indicated that you will make your data available on acceptance. We strongly recommend all authors decide on a data sharing plan before acceptance, as the process can be lengthy and hold up publication timelines. Please note that, though access restrictions are acceptable now, your entire data will need to be made freely accessible if your manuscript is accepted for publication. This policy applies to all data except where public deposition would breach compliance with the protocol approved by your research ethics board. If you are unable to adhere to our open data policy, please kindly revise your statement to explain your reasoning and we will seek the editor's input on an exemption. Please be assured that, once you have provided your new statement, the assessment of your exemption will not hold up the peer review process.

5. Please include captions for your Supporting Information files at the end of your manuscript, and update any in-text citations to match accordingly. Please see our Supporting Information guidelines for more information: http://journals.plos.org/plosone/s/supporting-information.

6. If the reviewer comments include a recommendation to cite specific previously published works, please review and evaluate these publications to determine whether they are relevant and should be cited. There is no requirement to cite these works unless the editor has indicated otherwise.

Additional Editor Comments:

Please note that we have only been able to secure a single reviewer to assess your manuscript. We are issuing a decision on your manuscript at this point to prevent further delays in the evaluation of your manuscript. Please be aware that the editor who handles your revised manuscript might find it necessary to invite additional reviewers to assess this work once the revised manuscript is submitted. However, we will aim to proceed on the basis of this single review if possible.

Could you please revise the manuscript to carefully address the concerns raised?

[Note: HTML markup is below. Please do not edit.]

Reviewer's Responses to Questions

**Comments to the Author**

1. Does the manuscript provide a valid rationale for the proposed study, with clearly identified and justified research questions?

Reviewer #1: Partly

**********

2. Is the protocol technically sound and planned in a manner that will lead to a meaningful outcome and allow testing the stated hypotheses?

Reviewer #1: Partly

**********

3. Is the methodology feasible and described in sufficient detail to allow the work to be replicable?

Reviewer #1: No

**********

4. Have the authors described where all data underlying the findings will be made available when the study is complete?

The PLOS Data policy

Reviewer #1: Yes

**********

5. Is the manuscript presented in an intelligible fashion and written in standard English?

Reviewer #1: Yes

**********

Please use the space provided to explain your answers to the questions above and, if applicable, provide comments about issues authors must address before this protocol can be accepted for publication. You may also include additional comments for the author, including concerns about research or publication ethics.

You may also provide optional suggestions and comments to authors that they might find helpful in planning their study.

Reviewer #1: This protocol proposes a scoping review on the use of user-centred design (UCD) and co-creation methods for digital health interventions in low-resource settings. While this topic is interesting overall, the manuscript has significant issues that should be addressed before publication. Please see below for more specific feedback.

Major:

1. The framing and motivation for the scoping review is not clear. In particular, in the introduction, it is not consistently clear that you are focusing on identifying papers that use UCD to design digital health interventions in low-resource settings. You mention digital health early on but then switch to talking about UCD and low-resource settings without digital health.

2. The introduction/framing of the paper only mentions UCD and co-creation but there are many other related methodologies such as co-design, participatory design, human-centred design (HCD), etc. You use the term HCD in line 105 but did not introduce it. The paper would benefit from clearly defining the term(s) you plan to use, and explaining how they relate to other similar methodologies, and explaining in your search strategy how you will capture as many of these as possible.

3. Intro, line 115-119: You have not made a strong case for why UCD and co-creation methodologies would need to be applied differently in a low-resource setting. The factors you list here could impact the success of digital health interventions, but not UCD methods. In fact, I would argue that UCD methods help to overcome these types of challenges in digital health in low-resource settings.

4. Line 148: Your third research question cannot be addressed with your planned methods. You would need to also identify studies that do not use UCD methods in order to compare them.

5. Methods: Your methods are not very clearly explained and are ambiguous in places. For example, the dates that will be included are not clear (some places say 10 year, some say 10-15). It would help to include an overview of the search strategy in the main paper, rather than the appendix (e.g., summarising the main search concepts and synonyms). It would also be helpful to have the databases that will be searched clearly listed (they are currently mentioned across different paragraphs, and a more detailed strategy is needed for Google Scholar which usually returns tens of thousands of results that cannot be systematically screened), as well as the inclusion/exclusion criteria. I would also suggest presenting your methods in the order they will be done. For example, you write that references of included articles will be screened before you have finished describing where the searches will take place. I see that you have checked these items off in the PRISMA checklist, but they did not feel very clear in the protocol.

6. It would be helpful to more clearly define if there are certain types of papers that will be included or excluded. Do they have to measure outcomes to be included? What if they only use co-design methods to develop a digital health tool, but do not evaluate it? Do they need to measure the outcomes you have specifically listed? What if they only mention the use of “UCD” or “co-design” but do not explicitly describe the methods used?

Minor comments:

7. Intro: in some areas, this protocol focuses on infectious disease specifically, though this does not seem to be part of the scoping review topic or strategy. I would suggest instead starting the introduction by talking about the burden of preventable diseases in low-resource settings, rather than listing rare infectious diseases of poverty.

8. Intro: Similarly, many of the digital health examples provided are very infectious-disease specific. If you plan to review studies that include all types of digital health tools, I think these examples should be expanded to include those that address health behaviours, chronic diseases, etc. as these are a huge area as well.

9. Line 146: I think this question needs to include “in low-resource settings.”

10. Please clarify how you will you define “low-resource” or “underserved” in high-income countries as part of your inclusion/exclusion criteria.

11. Types of studies: it is not typical to include systematic/scoping reviews in your results, but instead to check their references for relevant studies.

12. Line 175: you mentioned a restriction of 10 years in your abstract but state in Line 175 that you will just “prioritise” publications in 10-15 years but not restrict them. Since this is a systematic search and screening, there is no scope to prioritise – you either need to include the papers or not. Please also choose between 10 or 15 years. If you decide to restrict the strategy, please provide a justification.

13. Search strategy: The search strategy needs refinement – for example, you are missing the other spelling of “human-centred design” and many digital health-relevant terms like electronic medical records, electronic health records, chatbot, etc.

14. Ethics: scoping reviews do not require ethics approval.

15. Extraction table (appendix): it would also be helpful to extract data on the any specific framework that was used by the researchers (e.g., the Double Diamond, IDEAS framework, etc.). This also seems closely related to your first review question.

**********

what does this mean?). If published, this will include your full peer review and any attached files.

If you choose “no”, your identity will remain anonymous but your review may still be made public.

**Do you want your identity to be public for this peer review?**  For information about this choice, including consent withdrawal, please see our Privacy Policy

Reviewer #1: No

**********

[NOTE: If reviewer comments were submitted as an attachment file, they will be attached to this email and accessible via the submission site. Please log into your account, locate the manuscript record, and check for the action link "View Attachments". If this link does not appear, there are no attachment files.]

To ensure your figures meet our technical requirements, please review our figure guidelines: https://journals.plos.org/plosone/s/figures

You may also use PLOS’s free figure tool, NAAS, to help you prepare publication quality figures: https://journals.plos.org/plosone/s/figures#loc-tools-for-figure-preparation.

NAAS will assess whether your figures meet our technical requirements by comparing each figure against our figure specifications.

# Author comment: Author response to Decision Letter 1

**DOI:** 10.1371/journal.pone.0337671.r002

23 Jun 2026

Reviewer comment 1

The framing and motivation for the scoping review is not clear. In particular, in the introduction, it is not consistently clear that you are focusing on identifying papers that use UCD to design digital health interventions in low-resource settings. You mention digital health early on but then switch to talking about UCD and low-resource settings without digital health.

Response: We agree that the introduction did not consistently hold the three core concepts; UCD/co-creation, digital health and diagnostics, and low-resource settings in view simultaneously, drifting into a generic account of UCD. We have rethreaded the introduction so the specific intersection that defines the review is foregrounded throughout, re-anchoring the UCD and co-creation paragraphs to digital-health/diagnostic examples in low-resource settings, and adding an explicit scope statement.

Action taken: Revised the Introduction and Rationale to re-anchor each design paragraph to digital health/diagnostics in low-resource settings and added an explicit closing scope sentence specifying that the review focuses on studies applying UCD/co-creation to the design of digital health and diagnostic innovations in low-resource settings. The objective and review questions were aligned to mirror this framing.

Reviewer comment 2

The introduction/framing of the paper only mentions UCD and co-creation but there are many other related methodologies such as co-design, participatory design, human-centred design (HCD), etc. You use the term HCD in line 105 but did not introduce it. The paper would benefit from clearly defining the term(s) you plan to use, and explaining how they relate to other similar methodologies, and explaining in your search strategy how you will capture as many of these as possible.

Response: We agree. We have added a short terminology paragraph defining the two organizing constructs we adopt (UCD and co-creation), naming the adjacent methodologies (HCD, design thinking, co-design, co-production, participatory design, community-based participatory research), and explaining how they relate as a partly overlapping continuum of user involvement. We introduce HCD explicitly at first use and clarify that our search deliberately captures the full terminological family.

Action taken: Added a terminology/conceptual-scope paragraph to the Introduction; introduced “human-centered design (HCD)” at first use (tracked change); and clarified in the Search strategy and Appendix I that Concept Block 1 is constructed to capture the full family of related terms regardless of the label authors use.

Reviewer comment 3

Intro, line 115-119: You have not made a strong case for why UCD and co-creation methodologies would need to be applied differently in a low-resource setting. The factors you list here could impact the success of digital health interventions, but not UCD methods. In fact, I would argue that UCD methods help to overcome these types of challenges in digital health in low-resource settings.

Response: We fully accept this point, which has strengthened our argument. We have rewritten the rationale so that contextual factors (infrastructure, literacy, connectivity, socio-cultural diversity) are no longer presented as obstacles to the methods, but as precisely the realities UCD and co-creation are suited to navigate. The genuine gap is relocated to the evidence base: the lack of a consolidated picture of how these methods are applied in low-resource settings and with what outcomes.

Action taken: Rewrote the Rationale passage (tracked change): the deleted clause framing contextual factors as impeding the methods is replaced with text positioning UCD/co-creation as well suited to these settings and locating the gap in the under-consolidated evidence on application and outcomes.

Reviewer comment 4

Your third research question cannot be addressed with your planned methods. You would need to also identify studies that do not use UCD methods in order to compare them.

Response: The reviewer is correct. A comparison with non-co-creative approaches would require including comparator studies outside our inclusion criteria and beyond the descriptive intent of a scoping review. We have revised RQ3 to remove the explicit comparison and to focus on characterizing the outcomes reported within included UCD/co-creation studies, including how they are measured and which are sustained. Any within-study comparisons that appear will be summarized narratively.

Action taken: Revised RQ3 (tracked change) to: “What outcomes intended and unintended are reported in association with UCD and co-creation in digital health and diagnostic innovations in low-resource settings, how are these outcomes measured and reported?, and which are sustained over time?” The comparison clause was deleted.

Reviewer comment 5

Methods: Your methods are not very clearly explained and are ambiguous in places. For example, the dates that will be included are not clear (some places say 10 year, some say 10-15). It would help to include an overview of the search strategy in the main paper, rather than the appendix (e.g., summarising the main search concepts and synonyms). It would also be helpful to have the databases that will be searched clearly listed (they are currently mentioned across different paragraphs, and a more detailed strategy is needed for Google Scholar which usually returns tens of thousands of results that cannot be systematically screened), as well as the inclusion/exclusion criteria. I would also suggest presenting your methods in the order they will be done. For example, you write that references of included articles will be screened before you have finished describing where the searches will take place. I see that you have checked these items off in the PRISMA checklist, but they did not feel very clear in the protocol.

Response: We have revised the Methods comprehensively: standardized the eligibility window to 1 January 2015 – 31 December 2025; added a main-text overview of the three search concept blocks with representative synonyms (full strings in Appendix I); consolidated all databases and supplementary sources into a single list; specified a bounded, reproducible Google Scholar procedure; added explicit inclusion and exclusion criteria; and reordered the stages into execution sequence, moving reference-list/citation searching to after the database searches.

Action taken: Edited the abstract and Methods date language to a single firm window (tracked changes); corrected the framework attribution (see below); added the Types-of-studies/eligibility rewrite; and removed the duplicated synthesis section. Note: this comment also surfaced two latent inconsistencies we corrected the framework was mis-attributed (“JBI methodology... as updated by Levac et al.”; Levac et al. extends the Arksey & O’Malley framework, not JBI), now corrected to “JBI methodology for scoping reviews” with reporting per PRISMA-ScR; and the document contained two near-identical synthesis sections (“Data synthesis and presentation” and “Data analysis and presentation”), the duplicate of which has been deleted. Additionally we have included an overview of the search strategy within the text and specified the strategy for search for google scholar.

Reviewer comment 6

Do they have to measure outcomes to be included? What if they only use co-design methods... but do not evaluate it?... What if they only mention the use of “UCD” or “co-design” but do not explicitly describe the methods used?

Response: As a direct response, no, outcomes do not have to be measured. We have added explicit decision rules: (i) outcome measurement is not required for inclusion, since the review maps application; (ii) design-only studies are included, with the development stage recorded at extraction; (iii) the listed outcomes are illustrative of what we extract where present, not an inclusion threshold; and (iv) studies that merely name a label without describing any user-involvement activity are excluded, per a minimum-activity operational definition.

Action taken: Added the eligibility rules to the Types-of-studies/eligibility text (tracked change), including the minimum user-involvement-activity threshold and the explicit statement that formal evaluation is not required. The abstract inclusion wording was softened from “report on outcomes” to “where reported, outcomes” to remove the contradiction.

Reviewer comment 7

Intro: in some areas, this protocol focuses on infectious disease specifically, though this does not seem to be part of the scoping review topic or strategy. I would suggest instead starting the introduction by talking about the burden of preventable diseases in low-resource settings, rather than listing rare infectious diseases of poverty.

Response: We agree. We have rewritten the opening to motivate the review through the broader burden of preventable disease (communicable and non-communicable) and the systemic diagnostic/access gaps that digital health can help address. Specific infectious diseases are retained only as brief illustrations of digital diagnostic application, not as a statement of scope.

Action taken: Rewrote the opening paragraph (tracked change) to lead with the burden of preventable disease across communicable and non-communicable conditions and corrected the definitional error “non-infectious diseases (NCDs)” to “non-communicable diseases (NCDs).”

Reviewer comment 8

Intro: Similarly, many of the digital health examples provided are very infectious-disease specific. If you plan to review studies that include all types of digital health tools, I think these examples should be expanded to include those that address health behaviours, chronic diseases, etc. as these are a huge area as well.

Response: We agree and have broadened the illustrative examples to span non-communicable and chronic-disease management, health-behavior change and prevention, maternal and reproductive health, and mental health, alongside the existing infectious-disease diagnostic examples, presented explicitly as illustrations of the breadth the review will map.

Action taken: Expanded the digital-health example paragraphs (reflected in the broadened Introduction framing). We also corrected the word-order error “innovations digital health” to “digital health innovations” (tracked change), and flag for the editor that reference [6] supporting a point-of-care accuracy claim is a non-peer-reviewed source we are replacing.

Reviewer comment 9

Please clarify how you will you define “low-resource” or “underserved” in high-income countries as part of your inclusion/exclusion criteria.

Response: We have added an operational definition with two pathways: (i) LMICs classified using the World Bank income classification for the study year; and (ii) resource-constrained or underserved populations/sub-national areas within high-income countries, evidenced by indicators such as rural/remote populations, designated medically underserved areas, displaced or migrant populations, indigenous communities, or author-defined underserved groups, with a materiality requirement and reviewer adjudication for ambiguous cases.

Action taken: Added the operational definition to the eligibility criteria; corrected the extraction instrument (Appendix II, Section B) income-level options, where “Lower middle income” was duplicated and “Low income” omitted the first instance is changed to “Low income” (tracked change) and added a field recording the basis for the low-resource/underserved classification.

Reviewer comment 10

…it is not typical to include systematic/scoping reviews in your results, but instead to check their references for relevant studies.

Response: We agree. Existing reviews are no longer included as primary results; their reference lists will be screened to identify relevant primary studies, and they may inform background/contextual discussion. This avoids double-counting underlying primary studies.

Action taken: Revised the Types-of-studies criterion (tracked change) to exclude reviews as records while retaining them for reference-list screening and background; added a citation-screening step after the database searches; and removed “Review” from the extractable study-design options in Appendix II.

Reviewer comment 11

You mentioned a restriction of 10 years in your abstract but state... that you will just “prioritize” publications in 10-15 years but not restrict them... Please also choose between 10 or 15 years. If you decide to restrict the strategy, please provide a justification.

Response: We agree on both points. We have removed all “prioritization” language and adopted a single firm window of 1 January 2015 – 31 December 2025 (a 10-year period), applied as a hard database-level limit and enforced consistently throughout, with a justification added.

Action taken: Replaced the “no strict restriction... prioritizing... last 10–15 years” sentence with a firm 2015–2025 inclusion rule and justification (tracked change), and aligned the abstract date language (“within the last 15 years” → “between 2015 and 2025”). All other date references were reconciled.

Reviewer comment 12

Search strategy: The search strategy needs refinement. You are missing the other spelling of “human-centered design” and many digital health-relevant terms like electronic medical records, electronic health records, chatbot, etc.

Response: We have systematically revised Appendix I to include British/American and hyphenation variants for every design term (including both spellings of “human-centered/centered design”) and substantially expanded the digital-health block with the missing concepts electronic health/medical records (EHR/EMR), clinical decision support, chatbot/conversational agent, artificial intelligence/machine learning, remote/digital monitoring, wearables/sensors, and digital/computational diagnostics, among others. We have added a statement that the strategy will be peer-reviewed using the PRESS checklist before execution.

Action taken: Revised Appendix I (Concept Blocks 1 and 2) to add spelling/hyphenation variants and the additional digital-health terms; corrected typographical errors in Block 1 (“percon-centered”, unmatched bracket) and inserted explicit Boolean operators between synonym groups; and added a PRESS peer-review statement to the Methods. The main overview now reflects the expanded terms.

Reviewer comment 13

It would also be helpful to extract data on any specific framework that was used by the researchers (e.g., the Double Diamond, IDEAS framework, etc.). This also seems closely related to your first review question.

Response: We agree and have added a dedicated extraction field for the specific named framework/model applied (distinct from the broad methodology), seeded with common options (Double Diamond, Design Thinking/d.school, ISO 9241-210, IDEAS, Stanford Biodesign, EBCD, CeHRes Roadmap, etc.) plus “Other (specify)” and “None named.” We have noted in the synthesis that the distribution of these frameworks will be analyzed to answer RQ1.

Action taken: Added the “Specific design framework/model used” field to Appendix II, Section D; sharpened RQ1 to reference frameworks/models with examples (tracked change); and linked the new field to RQ1 in the Data synthesis section.

Reviewer comment 14

Ethics: scoping reviews do not require ethics approval.

Response: We agree that a literature-based scoping review does not require research-ethics approval. We have clarified this in the Ethics and dissemination section and reframed the institutional and national approvals as program-level (DI-DIDA) context reported for completeness, while retaining the dissemination and protocol-amendment statements.

Action taken: Added a leading sentence to the Ethics section (tracked change) stating that the review does not in itself require ethics approval, with the SU-ISERC/NACOSTI approvals reframed as broader-project context.

Reviewer comment 15

I think this question needs to include “in low-resource settings.” At Line 146

Response: We agree and have added the scope clause. We also confirmed that all three review questions are now consistently bounded to low-resource settings and that the phrasing “low

Attachment Submitted filename: Response to reviewers_UCD PROTOCOL.docx

# Peer review: Decision Letter 1

**DOI:** 10.1371/journal.pone.0337671.r003

**Authors:** Alejandro Mac Cawley

4 Aug 2026

Dear Dr. Otukpa,

Thank you for submitting your manuscript to PLOS ONE. After careful consideration, we feel that it has merit but does not fully meet PLOS ONE’s publication criteria as it currently stands. Therefore, we invite you to submit a revised version of the manuscript that addresses the points raised during the review process.

Please submit your revised manuscript by Sep 18 2026 11:59PM. If you will need more time than this to complete your revisions, please reply to this message or contact the journal office at plosone@plos.org. When you're ready to submit your revision, log on to https://www.editorialmanager.com/pone/ and select the 'Submissions Needing Revision' folder to locate your manuscript file.

- A letter that responds to each point raised by the academic editor and reviewer(s). You should upload this letter as a separate file labeled 'Response to Reviewers'.
- A marked-up copy of your manuscript that highlights changes made to the original version. You should upload this as a separate file labeled 'Revised Manuscript with Track Changes'.
- An unmarked version of your revised paper without tracked changes. You should upload this as a separate file labeled 'Manuscript'.

If you would like to make changes to your financial disclosure, please include your updated statement in your cover letter. Guidelines for resubmitting your figure files are available below the reviewer comments at the end of this letter.

If applicable, we recommend that you deposit your laboratory protocols in protocols.io to enhance the reproducibility of your results. Protocols.io assigns your protocol its own identifier (DOI) so that it can be cited independently in the future. For instructions see: https://journals.plos.org/plosone/s/submission-guidelines#loc-laboratory-protocols. Additionally, PLOS ONE offers an option for publishing peer-reviewed Lab Protocol articles, which describe protocols hosted on protocols.io. Read more information on sharing protocols at https://plos.org/protocols?utm_medium=editorial-email&utm_source=authorletters&utm_campaign=protocols.

As the corresponding author, your ORCID iD is verified in the submission system and will appear in the published article. PLOS supports the use of ORCID, and we encourage all coauthors to register for an ORCID iD and use it as well. Please encourage your coauthors to verify their ORCID iD within the submission system before final acceptance, as unverified ORCID iDs will not appear in the published article. *Only*  the individual author can complete the verification step; PLOS staff *cannot*  verify ORCID iDs on behalf of authors.

We look forward to receiving your revised manuscript.

Kind regards,

Alejandro F. Mac Cawley, Ph.D.

Academic Editor

PLOS One

Journal Requirements:

If the reviewer comments include a recommendation to cite specific previously published works, please review and evaluate these publications to determine whether they are relevant and should be cited. There is no requirement to cite these works unless the editor has indicated otherwise.

Please review your reference list to ensure that it is complete and correct. If you have cited papers that have been retracted, please include the rationale for doing so in the manuscript text, or remove these references and replace them with relevant current references. Any changes to the reference list should be mentioned in the rebuttal letter that accompanies your revised manuscript. If you need to cite a retracted article, indicate the article’s retracted status in the References list and also include a citation and full reference for the retraction notice.

Additional Editor Comments:

The reviewers have raised some remaining issues that should be addressed.

Reviewers' comments:

Reviewer's Responses to Questions

**Comments to the Author**

1. Does the manuscript provide a valid rationale for the proposed study, with clearly identified and justified research questions?

Reviewer #1: Partly

Reviewer #2: Yes

Reviewer #3: Yes

**********

2. Is the protocol technically sound and planned in a manner that will lead to a meaningful outcome and allow testing the stated hypotheses?

Reviewer #1: Partly

Reviewer #2: Yes

Reviewer #3: Yes

**********

3. Is the methodology feasible and described in sufficient detail to allow the work to be replicable?

Reviewer #1: No

Reviewer #2: Yes

Reviewer #3: Yes

**********

4. Have the authors described where all data underlying the findings will be made available when the study is complete?

The PLOS Data policy

Reviewer #1: Yes

Reviewer #2: Yes

Reviewer #3: Yes

**********

5. Is the manuscript presented in an intelligible fashion and written in standard English?

Reviewer #1: Yes

Reviewer #2: Yes

Reviewer #3: Yes

**********

Please use the space provided to explain your answers to the questions above and, if applicable, provide comments about issues authors must address before this protocol can be accepted for publication. You may also include additional comments for the author, including concerns about research or publication ethics.

You may also provide optional suggestions and comments to authors that they might find helpful in planning their study.

Reviewer #1: The authors have responded to many of my earlier comments and the manuscript is improved. However, there are still some remaining issues that should be addressed in order for this protocol to be clearly reported, rigorous, and replicable. Please see below for my additional feedback:

1. Line 59: Given the global focus of the review, the frequent mentions of SSA in the Intro don’t make sense. I would suggest moving away from SSA and describing challenges across LMICs more broadly.

2. Intro: The introduction still does not define HCD or discuss it in relation to other defined concepts like UCD or co-creation (Line 172). The justification for the focus on UCD and co-creation is also not very strong – why have UCD and co-creation been chosen as the focus, but not HCD or any other participatory methods? The framing needs to be very clear – if all of these methods are included in the review, then more elaboration is needed on the group of methods as a whole, rather than only highlighting two approaches. You could consider replacing “UCD and co-creation” throughout the paper with “UCD and related methodologies” or similar.

3. Line 166: there is a typo where what should be one sentence has been broken into multiple sentences.

4. Line 191: questions 1 and 2 do not mention UCD, though this was one of the main methodologies identified in the Intro. Again, it would be helpful to define a specific focus (I would advocate for including all participatory approaches together for a scoping review of this nature), then to be sure it is consistently applied across the research questions, search methods, etc.

5. Line 203: couldn’t “diagnostic innovations” already fit under the umbrella of digital health? It feels like you are mentioning a sub-area of digital health somewhat randomly, and only in some places of the protocol. This also applies to the title.

6. Line 210: From “Particularly…” onwards - you do not need to talk about these aspects of the inclusion criteria in the “population/setting” section. They fit better in later sections.

7. Line 233: I would argue that including papers that explicitly state they have used these methods but without describing the relevant activities should be included, given your point in the introduction about this being a problem in the field. This seems that it would then be a finding of your review if some proportion of the papers mention UCD/HCD/co-design, etc. but do not explicitly describe their methods.

8. Line 242: Search should not stop at 2025, but should go until the present

9. Line 251: reference needed for JBI

10. Line 260: do you mean “peer-reviewed literature” or “academic literature” and “grey literature” instead of “published” and “unpublished”?

11. Line 276: I don’t think #6 should be included in your search – this could exclude relevant papers that refer to high-resource settings, or papers in high-income countries that focus on low-resource communities or populations. Similarly, if you want to include papers focused on underserved communities in high-income settings, as stated in your Methods, you should include search terms related to these populations (e.g., underserved, low-income, marginalised, etc.)

12. Line 305: the editor can make the final call here, but I don’t think you need to mention these ethics approvals – they are not relevant for this scoping review and just take up unnecessary space, in my opinion.

13. Line 318: it appears that this search has already been conducted, but this is concerning as I have identified issues with the search terms that I think need to be corrected before the scoping review is completed.

14. Line 493: You should state in the protocol where the list of LMICs came from (e.g. World Bank classification and year) – this is currently only in your response to my comment, but not in the Methods.

15. Line 271: “In several electronic databases” is vague - it is still not very clear which databases will be searched. It is also not yet clear how grey literature will be located, as “grey literature sources” is quite vague too.

16. Appendix: you are missing search terms with the British spelling of “human-centred” and “user-centred”

Reviewer #2: - The introduction is well structured and clearly motivates the review. However, some technology-specific examples (e.g., malaria diagnostics, wearable monitoring, and point-of-care technologies) are described in greater detail than is necessary for a protocol. As the review focuses on UCD and co-creation rather than digital health technologies themselves, these examples could be condensed to place greater emphasis on the conceptual rationale and the implementation challenges motivating the review.

- The operational definition of “low-resource settings” requires further clarification. The eligibility criteria currently include LMICs as well as rural or underserved areas in high-income countries, but it is not clear why the latter are included or how they will be identified during screening. Rurality or underserved status does not necessarily equate to a low-resource setting. The authors should either (i) adopt a standard definition (e.g., World Bank LMIC classification), or (ii) provide a clear conceptual and operational justification for extending the review to resource-constrained populations within high-income countries, including explicit eligibility criteria.

- The “Study status and timeline” section appears to contain projected dates that have now passed. Please update this section to reflect the current status of the review (e.g., whether the literature search, screening, or data extraction have been completed). If the review has progressed since the protocol was first submitted, it would be helpful to clarify that the protocol continues to reflect the methods that were followed, and that any deviations will be reported in the final review.

- RQ2 asks: “How do co-creation processes influence…”. But, a scoping review maps reported evidence rather than demonstrating causal influence. A more cautious wording could be: “How are co-creation processes reported to influence…”.

- limitations may also acknoledge language restriction (English/French), and the anticipated heterogeneity of interventions, co-creation approaches, and reported outcomes, which may limit the comparability of findings.

Reviewer #3: I have carefully examined both the authors' response to the previous reviewer and the revised manuscript. The authors have made substantial efforts to address the concerns raised, and the manuscript has improved considerably. In particular, the scope of the review is now more clearly defined, the rationale is better aligned with the stated objectives, the review questions are more appropriate for a scoping review methodology, and the methodological description is more transparent and reproducible. The revised protocol is generally well organised and follows accepted guidance for scoping reviews.

Overall, these revisions adequately address the substantive concerns raised by the previous reviewer.

I only have a small number of minor observations before acceptance.

1. The Introduction remains somewhat lengthy- Although the broader examples of digital health have improved the manuscript, several sections describing individual technologies (for example wearable technologies, disease-specific examples and technical descriptions) appear more detailed than is necessary for a protocol. Some condensation would improve readability without affecting the scientific contribution. This is an editorial suggestion rather than a methodological concern.

2. The rationale could be slightly more concise- While the rationale is now much stronger, there remains some repetition around the value of documenting UCD/co-creation and the need for evidence mapping. Streamlining this section would improve flow.

3. A final proofread would be beneficial- I noticed occasional grammatical and stylistic inconsistencies (for example punctuation, sentence flow and wording) that would benefit from careful copy-editing prior to publication. These do not affect the scientific content but would improve readability.

The authors have responded appropriately and comprehensively to the previous review. The remaining observations are minor editorial suggestions rather than substantive methodological concerns. I therefore recommend acceptance after minor editorial revisions.

**********

what does this mean?). If published, this will include your full peer review and any attached files.

If you choose “no”, your identity will remain anonymous but your review may still be made public.

**Do you want your identity to be public for this peer review?**  For information about this choice, including consent withdrawal, please see our Privacy Policy

Reviewer #1: No

Reviewer #2: No

Reviewer #3: **Yes:**  Maneesh Kumar

**********

[NOTE: If reviewer comments were submitted as an attachment file, they will be attached to this email and accessible via the submission site. Please log into your account, locate the manuscript record, and check for the action link "View Attachments". If this link does not appear, there are no attachment files.]

To ensure your figures meet our technical requirements, please review our figure guidelines: https://journals.plos.org/plosone/s/figures

You may also use PLOS’s free figure tool, NAAS, to help you prepare publication quality figures: https://journals.plos.org/plosone/s/figures#loc-tools-for-figure-preparation.

NAAS will assess whether your figures meet our technical requirements by comparing each figure against our figure specifications.

# Author comment: Author response to Decision Letter 2

**DOI:** 10.1371/journal.pone.0337671.r004

10 Aug 2026

We thank the Editor and the three Reviewers for the time and care taken with this protocol. The comments were detailed and constructive and addressing them has led to substantive improvements in the coherence and reproducibility of the review in particular to the framing of the design methodologies under study, the internal consistency of the scope statement, the eligibility criteria, the search strategy, and the accuracy with which we report the status of the work.

Below we respond to each of the comments in turn. Reviewers' comments are reproduced verbatim; each response is followed in blue by a description of the corresponding changes to the manuscript. Revised text is quoted where it aids review. In the manuscript itself, all changes are marked using tracked changes. Where two or more Reviewers raised related points and in one instance where their recommendations pulled in opposite directions we have addressed them together and flagged the connection, so that the coherence of our revisions can be assessed across the reviews as a whole.

Reviewer #1: The authors have responded to many of my earlier comments and the manuscript is improved. However, there are still some remaining issues that should be addressed for this protocol to be clearly reported, rigorous, and replicable. Please see below for my additional feedback:

1. Line 59: Given the global focus of the review, the frequent mentions of SSA in the Intro don’t make sense. I would suggest moving away from SSA and describing challenges across LMICs more broadly.

Thank you for this keen observation. On a re-examination, we do fine the framing of the Introduction inconsistent with the scope of the review, which is defined at the level of low-resource settings rather than any single region. The repeated references to sub-Saharan Africa were a legacy of the regional focus of the parent DI-DIDA project and were not intended to narrow the review.

We have revised the Introduction to describe the disease burden, health-system constraints, and diagnostic gaps across low- and middle-income countries and other low-resource settings more broadly. Sub-Saharan Africa is retained only where a cited source reports region-specific data, and in those instances, it is presented as an illustrative example rather than as the framing of the review. We have also checked the Rationale and Inclusion criteria to confirm that the population and setting framing is now consistent throughout. Specifically;

• Introduction, paragraph 2: burden and health-system text re-cast at LMIC level. Revised text reads:

“These conditions account for a substantial share of deaths worldwide, with the burden concentrated in low- and middle-income countries (LMICs) [3]. This is partly a consequence of persistent health-system constraints in these settings — including infrastructure, financing, and human-resource limitations — that restrict the adoption and impact of medical and diagnostic technologies [4].”

• Point-of-care testing paragraph: both references to sub-Saharan Africa replaced with “low-resource settings” and “these settings”; the sentence fragment beginning “Ensuring the relatively accurate testing…” merged into the preceding sentence.

• Reference [4] (a sub-Saharan Africa–specific analysis) is now signaled as regional evidence and paired with an LMIC-wide source supporting the general claim.

• A sentence has been added stating that “low-resource settings” as used in this review includes underserved populations within high-income countries, consistent with the Inclusion criteria and with the revised search strategy.

2. Intro: The introduction still does not define HCD or discuss it in relation to other defined concepts like UCD or co-creation (Line 172). The justification for the focus on UCD and co-creation is also not very strong – why have UCD and co-creation been chosen as the focus, but not HCD or any other participatory methods? The framing needs to be very clear – if all of these methods are included in the review, then more elaboration is needed on the group of methods as a whole, rather than only highlighting two approaches. You could consider replacing “UCD and co-creation” throughout the paper with “UCD and related methodologies” or similar.

We appreciate the Reviewer for pressing us on this point, which has led to a clearer and more defensible framing of the review. Our intention was never to exclude HCD, co-design, participatory design, or design thinking: our search strategy already contains terms for each of these, and our operational definition of eligibility turns on the user-involvement activities a study describes rather than on the label it applies. The framing in the Introduction did not reflect this. The Reviewer is correct that HCD was invoked without definition and that our emphasis on two named approaches was inconsistent with an eligibility criterion that is, in practice, method-agnostic.

We have addressed the comment in three ways. First, we have added a paragraph to the Introduction defining the family of participatory and user-involving design methodologies UCD, HCD, co-creation, co-design, participatory design, and design thinking setting out what they share (iterative development grounded in users’ contexts) and where they differ (principally in the depth of user involvement, from consultation through to equitable partnership), supported by a new table of working definitions. Second, we have adopted the umbrella phrasing the Reviewer suggests, using “UCD and related participatory design methodologies” as the framing term and reserving specific labels for places where a specific approach is meant. Third, we have made explicit that we do not treat these labels as reliable markers of practice: because the terminology is applied inconsistently, eligibility decisions rest on the activities a study describes, and the author-applied label is recorded as an extraction variable so that terminological variation becomes a finding of the review rather than a filter upon it. Specifically;

• Introduction: the three-sentence terminology passage replaced with a full definitional paragraph covering UCD, HCD, participatory design, co-design, co-creation, and design thinking, and the continuum of user involvement that distinguishes them. HCD is now defined at first use.

• New Table 1 added (“Working definitions of user-involving design approaches”), with columns for term, working definition, distinguishing emphasis, and key source; the search strategy table renumbered accordingly.

• Umbrella phrasing adopted throughout the title, Abstract, Rationale, Objectives, review questions, Inclusion criteria, Data extraction, Data synthesis, and Strengths and limitations.

• Rationale: justification for covering the family strengthened. Added text reads:

“We include the full family of user-involving design approaches rather than a single named method because the terminology is used inconsistently across this literature: studies applying substantively similar methods describe them variously, and studies invoking the same label may describe very different depths of user involvement. Restricting the review to one or two labels would therefore fragment a body of evidence that is methodologically continuous, and would systematically miss relevant work.”

• Inclusion criteria: a sentence added drawing the outer boundary of the family — approaches applied to the design or development of a digital health innovation are eligible; participatory approaches applied solely to service delivery or research governance, with no associated technology, are not.

• Appendix II, Section D: “Stated Methodology/Framework” converted from free text to a coded list (UCD / HCD / co-design / co-creation / participatory design / design thinking / CBPR / other / none stated), with a parallel free-text field capturing the authors’ verbatim wording.

3. Line 166: there is a typo where what should be one sentence has been broken into multiple sentences.

We appreciate that you pointed this out, such errors have the potential to confuse the reader and dampen the message. We have corrected this as appropriate.

4. Line 191: questions 1 and 2 do not mention UCD, though this was one of the main methodologies identified in the Intro. Again, it would be helpful to define a specific focus (I would advocate for including all participatory approaches together for a scoping review of this nature), then to be sure it is consistently applied across the research questions, search methods, etc.

We thank the Reviewer for this observation and for the accompanying recommendation, which we have adopted. Indeed, our review questions were not fully in line with the Introduction: questions 1 and 2 referred only to co-creation, question 3 to “UCD and co-creation”, while the eligibility criteria and search strategy were in fact broader than both.

Following the Reviewer’s advice, and consistent with our response to Comment 2, we have adopted the full family of participatory and user-involving design approaches as the explicit scope of the review and have rewritten the review questions so that this scope is applied uniformly. We have additionally conducted a consistency audit across the title, Abstract, Objectives, review questions, Inclusion criteria, search strategy, and data extraction instrument to confirm that a single scope statement is now used throughout, and have aligned the description of innovation types across all three questions, which was also previously inconsistent. Specifically:

• Review questions reworded. The revised questions read:

• What participatory and user-involving design approaches e.g. UCD, HCD, co-design, participatory design, and co-creation and which named frameworks, processes, or models (e.g. the Double Diamond, IDEAS, the ISO 9241-210 cycle) are applied in the development of digital health innovations for low-resource settings, and how does their use vary across regions, healthcare contexts, and technology types?

• How are participatory and user-involving design processes reported to influence the design and functionality of digital health innovations in low-resource settings — at which stages of the development lifecycle, using which methods, with which stakeholder groups, and at what depth of user involvement?

• What outcomes, intended and unintended (e.g. usability, acceptability, adoption, clinical accuracy, scalability, sustainability), are reported in association with these approaches in digital health innovations in low-resource settings, and how are these outcomes defined, measured, and reported?

******A further sub-question has been added in response to Comment 5: How completely are participatory design processes reported in this literature, and what proportion of studies invoke a named approach without describing the activities undertaken?

• Objectives section and Abstract (Objective and Inclusion criteria) revised to carry the same scope statement.

• Consistency audit completed across all sections naming the scope; the eligibility paragraph under “Types of studies”, which was already method-agnostic, is unchanged in substance.

• Formatting corrected: the review questions and the Inclusion criteria headings had been formatted as a single continuous numbered list and are now correctly structured.

5. Line 203: couldn’t “diagnostic innovations” already fit under the umbrella of digital health? It feels like you are mentioning a sub-area of digital health somewhat randomly, and only in some places of the protocol. This also applies to the title.

We thank the Reviewer for bringing this up and respectfully push back on the point. Digital diagnostics are indeed a sub-category of digital health, and our paired phrasing “digital health and diagnostics” only appeared redundant (not all diagnostic technologies have a digital component) however, our scope is limited to diagnostic innovations with a digital component for example smartphone-based microscopy, AI-assisted image analysis, connected point-of-care readers, and digitally enabled diagnostic decision support all of which fall within the definition of digital health we adopt. Purely analogue diagnostic tests without a digital component are not eligible.

We have therefore removed the paired construction throughout and now refer consistently to digital health innovations, and we have amended the title accordingly. To preserve transparency without reintroducing redundancy, we retain digital diagnostics as an explicitly named category within our taxonomy of eligible digital health interventions, and we state once, in the Rationale, that the review pays particular attention to diagnostic applications given the persistent diagnostic gap in low-resource settings and the framing of the wider DI-DIDA project. Innovation type is recorded at extraction, so the distribution of diagnostic and other digital health applications will be reported as a finding rather than asserted as a scope boundary. Specifically

• Title revised to remove “and diagnostics”; the revised title reads: “User-centred design and related participatory approaches in digital health innovation for low-resource settings: a scoping review protocol”.

• Paired phrasing removed from the Abstract (Objective and Methods), review question 3, the Data extraction section, and Strengths and limitations.

• Abstract, Introduction: the sentence narrowing the evidence gap to diagnostics rewritten to describe the gap across digital health innovation as a whole.

• Inclusion criteria, Context: the intervention list now names “digitally enabled diagnostics and point-of-care testing” alongside eHealth, mHealth, telehealth, and wearables, and an explicit exclusion has been added:

i. Diagnostic tests without a digital component (e.g. conventional lateral-flow rapid diagnostic tests read by eye) are not eligible unless the innovation includes a digital element such as automated reading, image capture and analysis, connectivity, or data capture.

ii. Rationale: one sentence added stating the review’s particular attention to diagnostic applications and the reason for it.

• Appendix II, Section C retitled “Digital Health Innovation (details)”; technology-type options reviewed to confirm each is unambiguously digital.

• Search strategy: the diagnostic terms are retained without a digital qualifier to preserve sensitivity; the Study selection section now states that the digital-component requirement is applied at screening.

6. Line 210: From “Particularly…” onwards - you do not need to talk about these aspects of the inclusion criteria in the “population/setting” section. They fit better in later sections.

This is a keen observation and important in the logical flow of the paper, thank you for highlighting it. It has been implemented.

7. Line 233: I would argue that including papers that explicitly state they have used these methods but without describing the relevant activities should be included, given your point in the introduction about this being a problem in the field. This seems that it would then be a finding of your review if some proportion of the papers mention UCD/HCD/co-design, etc. but do not explicitly describe their methods.

We thank the Reviewer for this insight, which we found persuasive. The Reviewer is correct that our exclusion rule was in tension with our own framing: having argued that inconsistent and superficial use of participatory design terminology is a recognized problem in this field, it was inconsistent to then remove from the review precisely those studies that exemplify it. We have revised the eligibility criteria so that studies claiming the use of a named participatory or user-involving design approach are eligible whether they describe the underlying activities.

A study is now eligible if it reports the development, adaptation, or evaluation of a digital health innovation in a low-resource setting and either (a) describes at least one concrete user-involvement activity, or (b) explicitly claims the use of a named user-involving design approach without describing the activities undertaken. We have added an extraction variable classifying each included study by the completeness of its reporting fully described, partially described, or label-only and will report this distribution

Attachment Submitted filename: Response to reviewers_UCD PROTOCOL_REVISED.docx

# Peer review: Decision Letter 2

**DOI:** 10.1371/journal.pone.0337671.r005

**Authors:** Alejandro Mac Cawley

19 Aug 2026

User-centered design and related participatory approaches in digital health innovations for low-resource settings: A scoping review protocol

PONE-D-25-59947R2

Dear Dr. Otukpa,

We’re pleased to inform you that your manuscript has been judged scientifically suitable for publication and will be formally accepted for publication once it meets all outstanding technical requirements.

Within one week, you’ll receive an e-mail detailing the required amendments. When these have been addressed, you’ll receive a formal acceptance letter and your manuscript will be scheduled for publication.

An invoice will be generated when your article is formally accepted. Please note, if your institution has a publishing partnership with PLOS and your article meets the relevant criteria, all or part of your publication costs will be covered. Please make sure your user information is up-to-date by logging into Editorial Manager at Editorial Manager® and clicking the ‘Update My Information' link at the top of the page. For questions related to billing, please contact billing support.

If your institution or institutions have a press office, please notify them about your upcoming paper to help maximize its impact. If they’ll be preparing press materials, please inform our press team as soon as possible -- no later than 48 hours after receiving the formal acceptance. Your manuscript will remain under strict press embargo until 2 pm Eastern Time on the date of publication. For more information, please contact onepress@plos.org.

Kind regards,

Alejandro F. Mac Cawley, Ph.D.

Academic Editor

PLOS One

Additional Editor Comments (optional):

I'm pleased to inform you that your manuscript has been deemed suitable for publication in PLOS One. Congratulations! Your manuscript is now being handed over to our production team.

Reviewers' comments:

Reviewer's Responses to Questions

**Comments to the Author**

1. Does the manuscript provide a valid rationale for the proposed study, with clearly identified and justified research questions?

Reviewer #2: Yes

**********

2. Is the protocol technically sound and planned in a manner that will lead to a meaningful outcome and allow testing the stated hypotheses?

Reviewer #2: Yes

**********

3. Is the methodology feasible and described in sufficient detail to allow the work to be replicable?

Reviewer #2: Yes

**********

4. Have the authors described where all data underlying the findings will be made available when the study is complete?

The PLOS Data policy

Reviewer #2: No

**********

5. Is the manuscript presented in an intelligible fashion and written in standard English?

Reviewer #2: Yes

**********

Please use the space provided to explain your answers to the questions above and, if applicable, provide comments about issues authors must address before this protocol can be accepted for publication. You may also include additional comments for the author, including concerns about research or publication ethics.

You may also provide optional suggestions and comments to authors that they might find helpful in planning their study.

Reviewer #2: I appreciate the authors' careful and comprehensive responses. The manuscript has improved substantially, particularly in the conceptual framing, operational definition of low-resource settings, clarification of the study status, refinement of the review questions, and transparency of the protocol limitations. I believe the major methodological concerns raised in the previous review have been satisfactorily addressed.

Before publication I recommend one final consistency review to ensure that terminology is used uniformly throughout, particularly for:UCD and related participatory design approaches, digital health innovations, low-resource settings etc.

**********

what does this mean?). If published, this will include your full peer review and any attached files.

If you choose “no”, your identity will remain anonymous but your review may still be made public.

**Do you want your identity to be public for this peer review?**  For information about this choice, including consent withdrawal, please see our Privacy Policy

Reviewer #2: No

**********

# Editor report: Acceptance letter

**DOI:** 10.1371/journal.pone.0337671.r006

**Authors:** Alejandro Mac Cawley

PONE-D-25-59947R2

PLOS One

Dear Dr. Otukpa,

I'm pleased to inform you that your manuscript has been deemed suitable for publication in PLOS One. Congratulations! Your manuscript is now being handed over to our production team.

At this stage, our production department will prepare your paper for publication. This includes ensuring the following:

* All references, tables, and figures are properly cited

* All relevant supporting information is included in the manuscript submission,

* There are no issues that prevent the paper from being properly typeset

You will receive further instructions from the production team, including instructions on how to review your proof when it is ready. Please keep in mind that we are working through a large volume of accepted articles, so please give us a few days to review your paper and let you know the next and final steps.

Lastly, if your institution or institutions have a press office, please let them know about your upcoming paper now to help maximize its impact. If they'll be preparing press materials, please inform our press team within the next 48 hours. Your manuscript will remain under strict press embargo until 2 pm Eastern Time on the date of publication. For more information, please contact onepress@plos.org.

You will receive an invoice from PLOS for your publication fee after your manuscript has reached the completed accept phase. If you receive an email requesting payment before acceptance or for any other service, this may be a phishing scheme. Learn how to identify phishing emails and protect your accounts at https://explore.plos.org/phishing.

If we can help with anything else, please email us at customercare@plos.org.

Thank you for submitting your work to PLOS One and supporting open access.

Kind regards,

PLOS One Editorial Office Staff

on behalf of

Dr. Alejandro F. Mac Cawley

Academic Editor

PLOS One
